# CtBP1 sustains activity-dependent muscle properties and dampens synaptic, contractile and metabolic changes triggered by denervation

**DOI:** 10.1186/s13395-026-00421-w

**Published:** 2026-03-23

**Authors:** Olivia Cattaneo, Gaetan Lopez, Jayasimman Rajendran, Florent Chabry, Nicolas Liaudet, Sergei Startchik, Alexandre Prola, Perrine Castets

**Affiliations:** 1 Department of Cell Physiology and Metabolism, Faculty of Medicine, University of Geneva, Geneva, CH-1211 Switzerland; 2 Bioimaging Core Facility, Faculty of Medicine, University of Geneva, Geneva, 1211 Switzerland

**Keywords:** CtBP1, Skeletal muscle, Denervation, Metabolism, Mitochondria, Fiber type

## Abstract

**Background:**

Denervation triggers dramatic atrophy of skeletal muscle, accompanied by synaptic, contractile and metabolic changes. Several factors were shown to contribute to genetic reprogramming and proteostasis changes after denervation. However, the mechanisms underlying the coordinated regulation of denervation-induced muscle fiber remodeling remain misunderstood.

**Methods:**

We investigated the role of the transcriptional co-repressor CtBP1 in the regulation of denervation-induced responses in muscle fibers. To this end, we analyzed its expression and localization in innervated and denervated muscles and assessed the consequences of its knockdown induced in vivo with AAV9, on synaptic, contractile and metabolic properties of muscle fibers.

**Results:**

CtBP1 was present both in sub- and non-synaptic myonuclei in innervated muscle. Although CtBP1 levels remained unchanged in denervated muscle, CtBP1 accumulated transiently in myonuclei after 2 days of denervation in TA/EDL muscles. *Ctbp1* knockdown perturbed the expression of a large set of activity-independent and -dependent genes in innervated and denervated skeletal muscles. Reducing CtBP1 levels had limited effect on the expression of most synaptic genes, but increased transcript levels of *Chrne*, encoding the adult ε sub-unit of acetylcholine receptors (AChR). However, it did not affect AChR turnover or maintenance of the post-synaptic compartment upon denervation. Importantly, we uncovered that *Ctbp1* knockdown exacerbates denervation-induced changes in metabolic gene expression, including most genes encoding proteins of the respiratory chain complexes. Consistently, it induced a contractile shift towards slower fibers in innervated fast muscle, mimicking denervation, and enhanced the denervation-induced metabolic transition towards oxidative slow-twitch fibers. Moreover, *Ctbp1* knockdown precipitated the profound ultrastructural remodeling of mitochondria network induced after denervation.

**Conclusions:**

Our study unveils that CtBP1 sustains the innervated muscle pattern and antagonizes the effect of denervation on synaptic, contractile and metabolic muscle properties, with important implications for CtBP1-related muscle diseases.

**Supplementary Information:**

The online version contains supplementary material available at 10.1186/s13395-026-00421-w.

## Background

Skeletal muscle contraction is controlled by motoneurons, which connect to myofibers at the neuromuscular junction (NMJ). Efficient neurotransmission is ensured by the accumulation of synaptic proteins, such as acetylcholine receptors (AChRs), specifically in the highly specialized, sub-synaptic region of myofibers (i.e., the endplate). Within each multinucleated myofiber, only the few myonuclei located at the endplate express synaptic genes, while synaptic gene expression is repressed in all other nuclei [1, 2]. Nerve injury quickly triggers a switch back to a more embryonic genetic program in myonuclei, which includes the re-expression of the myogenic transcription factor myogenin, and in turn, of synaptic genes in non-synaptic regions of denervated myofibers [3, 4]. Synaptic remodeling coordinates with long-term effects of denervation, which include severe muscle atrophy and a switch in the metabolic and contractile properties of myofibers. The class II histone deacetylase (HDAC) 9 [5] and the transcriptional repressors dachshund family transcription factor 2 (Dach2) [3], Y-box binding protein 3 (YBX3 or Msy3) [6] and C-terminal binding protein 1 (CtBP1) [7] have been involved in the repression of synaptic and myogenic gene expression in innervated muscle. In contrast, HDAC4 induction triggers denervation-induced release of their repression [8–10] and contributes to the up-regulation of atrophy-related genes and to the metabolic switch in denervated muscle [10–12]. Whether other factors contribute to metabolic and synaptic remodeling upon denervation remains largely unknown.

CtBP1 is a ubiquitous and evolutionarily conserved transcriptional regulator forming complexes with DNA-binding proteins through a PXDLS motif [13]. The *CTBP1* gene encodes a long isoform (CtBP1-L) and a short alternatively spliced isoform, known as Brefeldin A-ADP ribosylated substrate (BARS or CtBP1-S). While CtBP1-L was initially characterized as a transcriptional co-repressor, CtBP1-S was shown to regulate membrane dynamics by promoting fission of the Golgi tubular network [14–16]. These findings indicate that CtBP1 proteins can exert both nuclear and cytoplasmic functions. However, whether both isoforms contribute to these two functions depending on cellular context remains unclear. CtBP1 activity is regulated through post-translational modifications influencing its shuttling from cytoplasm to nuclei. In particular, p21-activated kinase 1 (Pak1), by phosphorylating CtBP1 on Ser158, sequesters it in the cytoplasm and inhibits its co-repressor activity, while favoring its fission activity [17, 18]. Pak1-dependent CtBP1 nuclear export in skeletal muscle contributes to the release of myogenic and synaptic gene repression 3 days after denervation [7]. Interestingly, mutations in *CTBP1* cause muscle dysfunction marked by dystrophic features and mitochondrial alterations as part of the *Hypotonia*,* Ataxia*,* Developmental Delay and Tooth-enamel* syndrome (HADDTS) [19–21]. While the oncogenic role of CtBP1 has been widely reported [22], the pathomechanisms underlying HADDTS-associated muscle dysfunction are largely unknown.

Here we examined the physiological roles of CtBP1 in regulating activity-dependent processes in skeletal muscle. We show that denervation triggers a quick and transient accumulation of CtBP1 in myonuclei of fast muscles. Knocking down *Ctbp1* mimicked in innervated muscle and exacerbated in denervated muscle the denervation-induced synaptic, contractile and metabolic changes, as well as the ultrastructural remodeling of mitochondria network. These findings indicate that CtBP1 sustains the innervated pattern by antagonizing the complex synaptic, contractile and metabolic changes triggered by denervation in skeletal muscle.

## Methods

### Animals

C57BL6 control mice were kept in a conventional animal facility with a fixed 12 h dark/light cycle at 23 °C. For denervation, a segment of around 5 mm of sciatic nerve was surgically removed, as previously described [23]. Upon denervation, muscle mass variation (%) was calculated as the difference of the mass of the denervated and innervated muscles (from the contralateral leg), normalized to the mass of the innervated muscle. AAV9 carrying shRNA against mouse *Ctbp1*, *Ctbp2* or scramble (Vector Biolabs) were injected in the anterior hind limb compartment (8.10^10^ virus particle). Mice were sacrificed 4 weeks after AAV injection. All animal studies were performed in accordance with the European Union guidelines for animal care and approved by the Veterinary Office of the Canton of Geneva (application number GE220/GE227).

### Cell culture

C2C12 cell line was acquired from ATCC (CRL-1772) and cultured in Dulbecco’s modified Eagle’s medium (DMEM – Sigma D5796) supplemented with 10% fetal bovine serum and 1% Penicillin-Streptomycin (Pen/Strp). Cell differentiation was induced by switching to DMEM with 2% horse serum and 1% Pen/Strep.

### Transcript expression analyses

Total RNA was extracted from TA muscle or cells using RNeasy Fibrous Tissue Mini Kit (Qiagen). DNAse-treated mRNA were reverse transcribed using High-Capacity cDNA Reverse Transcription Kit (ThermoFisher). Quantitative PCR were performed with Power Up Sybr Master Mix (ThermoFisher), analyzed with StepOne software, and normalized on *Tbp* expression. Primers are listed in Supplementary Table 1. For RNAseq, RNA quantification was performed with a Qubit fluorometer (ThermoFisher) and RNA integrity assessed with a Bioanalyzer (Agilent Technologies). RNA-seq libraries were prepared with TruSeq mRNA stranded kit and sequenced on the Illumina HiSeq 4000 sequencer according to the manufacturer’s instruction at the iGE3 platform (Geneva, Switzerland). Quality control of the dataset was checked using FastQC Version 0.11.9. The FASTQ files will be deposited at Gene Expression Omnibus upon acceptance. Sequence reads were trimmed for adaptor sequence using Trimmomatic, aligned to *M. musculus* genome GRCm39 in R, and transformed to count tables using Rsubread V.2.12.2 package from Bioconductor. Count data were used for differential expression gene (DEG) analysis using Limma V.3.54.1 package and edgR V.3.40.2 from Bioconductor. DEGs between two conditions were filtered based on |fold change| up or down ≥ 1.5 and statistical p-value < 0.05. PCA plot was generated using R packages (ggfortify, ggplot2, and stats). Venn diagram was created using Venny 2.1.0. Gene ontology analyses were performed using filtered DEG sets against the GeneOntology database using Fgsea V.1.24.0 from Bioconductor packages in R. Dot plots and Volcano plots were generated using the ggplot2 Version 3.4.0 package in R, and heatmaps for individual genes lists were generated using GraphPad Prism Version 9.

### Western blot analysis

Proteins were extracted from cells and nitrogen-powdered TA muscle with RIPA lysis buffer (50mM Tris HCl pH8, 150mM NaCl, 1% NP-40, 0.5% Sodium Deoxycholate, 0.1% SDS, 1% Triton X100, 10% Glycerol) supplemented with proteases (Pierce) and phosphatases inhibitors (Roche). Lysates were sonicated twice 10 s and centrifuged at 10’000 RPM for 10 min at 4 °C. Nuclear/cytoplasmic fractions were obtained as described by Di Mauro et al., using whole muscles that were briefly fixed with 4% paraformaldehyde (PFA) [24]. Protein lysates were dosed with the BCA Protein Assay (Pierce) and separated in polyacrylamide SDS gels, and transferred to nitrocellulose membrane. Membranes were blocked in TBS, 0.1% Tween 20, 3% BSA, and incubated successively with primary antibodies and HRP-coupled secondary antibodies. Proteins were revealed with LumiGLO Peroxidase Chemiluminescent Substrate Kit (Sera Care) and analyzed with the iBright1500 (Invitrogen).

### Inorganic staining and immunostaining

For histology, TA muscles were frozen in nitrogen-cooled isopentane. 8 μm sections were stained with Hematoxylin/Eosin (H&E) and for NADH dehydrogenase activity (NADH tetrazolium reductase reaction, NADH-TR), succinate dehydrogenase activity (SDH) and cytochrome c oxidase activity (COX), as previously described [25, 26]. Pictures were captured with a widefield Zeiss Axioskop 2 plus microscope. For immunostaining, TA sections and EDL muscles were fixed in 4% PFA and quenched in PBS, 0.1 M Glycine. TA sections were blocked in PBS, 3% IgG free BSA (Jackson Immunoresearch), 0.2% Triton X100. For fiber type staining, TA sections were unfixed and blocked in PBS, 3% IgG free BSA, with AffiniPure Mouse IgG Fab Fragments (Jackson Immunoresearch). EDL muscles were micro-dissected into bundles or single fibers and blocked in PSB, 3% BSA, 2% Triton X100. Sections, bundles and single fibers were incubated sequentially with primary and fluorescent secondary antibodies, then mounted in Vectashield with or without DAPI (Vector Laboratories). Fluorescent α-bungarotoxin was from Invitrogen (ThermoFisher Scientific). Pictures were acquired using fluorescent widefield Zeiss Axio Imager M2, confocal Axio Imager Z2 Basis LSM 800 or Leica TCS SP8 STED 3X microscope. Fiber type and size distribution was determined based on the laminin staining with Fiji, as previously described [27].

### AChRs turnover assay

AChR turnover was assessed by sequential injection of 25 pmoles of α-bungarotoxin-Alexa488 and -Alexa555 (Invitrogen), as previously done [28]. Pictures of whole-mount EDL muscle fiber bundles were acquired with a confocal Axio Imager Z2 Basis LSM 800. Pixel dominance corresponding to “old” and new AChRs and endplate volume were calculated using Fiji and MATLAB software.

### Antibodies

The following antibodies were used: CtBP1 (PA582651; 1/2000 for IF on sections, 1/250 for IF on fibers) from ThermoFisher; DRP1 (8570), DRP1^P637^ (20990), DRP1^P616^ (4494), Pak1 (2602), CtBP1 (8684; 1/200 for IF), GAPDH (2118) and OPA1 (80471) from Cell Signaling; Actinin (A7732) from Sigma; CtBP1 (612042; 1/500 for IF) from BD Biosciences; Tubulin (15246), OXPHOS kit (110413), Laminin (11575; 1/500 for IF), Pink1 (23707) and TOMM20 (186735; 1/250 for IF) from Abcam; Synaptophysin (GT2589; 1/250 for IF) from Genetex Lucerna; Myosin Heavy Chain IIB (BF-F3; 1/25 for IF), IIA (sc-71; 1/50 for IF), I (BA.D5; 1/50 for IF) from Developmental Studies Hybridoma Bank; MFN1 (66776-1) from Proteintech. All antibodies were diluted at 1/1000 for Western blot.

### Images analysis

Images were analyzed with Zen lite, LAS X, Fiji, Imaris and MATLAB software. For the analysis of mitochondria orientation, 3D confocal images of TOMM20 were analyzed with MATLAB R2024b. Briefly, the central frame of each image stack was preprocessed with an edge-preserving Gaussian bilateral filter. The fiber orientation was determined by applying morphological closing, followed by automatic segmentation using Otsu’s method. Mitochondria were enhanced using a Hessian-based multiscale Frangi vesselness filter, followed by automatic segmentation. The orientation angle of each mitochondrial object was then measured relative to the fiber orientation. To evaluate the volume of individual mitochondrial, 3D confocal images of TOMM20 were analyzed in Imaris 10.2.0 using the surfaces model generated through software’s machine learning-based segmentation methods. Mean fluorescence intensity of CtBP1 in nuclei and cytoplasm of isolated fibers was analyzed using Imaris 11.0.0. Segmentation was performed using the Surfaces module with machine-learning classification, based on the CtBP1 channel for individual fibers, the DAPI channel for nuclei, and both CtBP1 and DAPI channels for cytoplasmic regions.

### Statistical analysis

Results are expressed as mean ± SEM of independent samples, with *n* (number of individual experiments) ≥ 3. Statistical comparison was performed using two-tailed Student’s *t*-test, one/two-way ANOVA test, or Kolmogorov-Smirnov test, depending on the conditions. A 0.05 level of confidence was accepted for statistical significance.

## Results

### CtBP1 protein levels are independent from neural activity

**Fig. 1:**
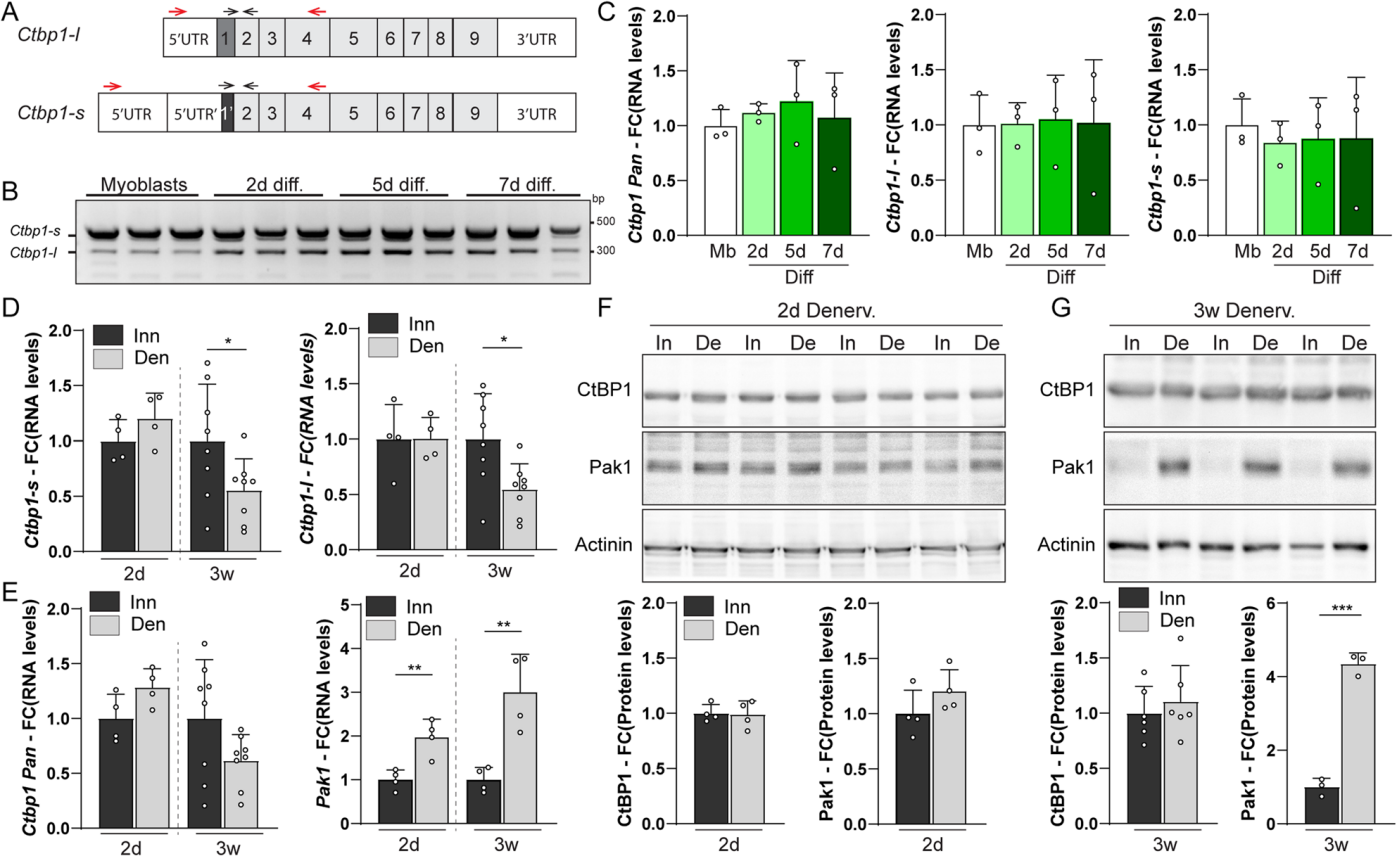
Expression of CtBP1-L and CtBP1-S upon muscle differentiation and denervation. **A** Organization of *Ctbp1-l* and *Ctbp1-s* mRNA in mouse. Exons and untranslated (UTR) sequences are represented by grey and white boxes, respectively. Primers used for PCR and qPCR are shown on top of mRNA with red and black arrows, respectively. **B** Expression of *Ctbp1-l* and *Ctbp1-s* is detected by PCR in C2C12 myoblasts and after 2 to 7 days (d) of differentiation (diff). *n* = 3 independent samples. **C** Transcript levels of total *Ctbp1*, *Ctbp1-l* and *Ctbp1-s* in C2C12 myoblasts (Mb) and after 2 to 7 days (d) of differentiation (diff). Levels are relative to *Tbp* mRNA and to myoblast. **D** and **E** Transcript levels of *Ctbp1-l* and *Ctbp1-s* (**D**) and of total *Ctbp1* and *Pak1* (**E**) in innervated (Inn) and denervated (Den – 2 days and 3 weeks) TA muscles. Levels are relative to *Tbp* mRNA and to innervated muscle. **F** and **G** Western Blot analysis of CtBP1 and Pak1 in innervated muscle (In) and 2 days (**F**) or 3 weeks (**G**) after denervation (De). Protein levels are normalized to Actinin and to innervated muscle. All values are mean ± s.d.; *n* = 3 biological replicates per group (**C**) and 4 (2 days **D-F**), 8 (*Ctbp1*, 3 weeks **D**-**E**), 4 (*Pak1*, 3 weeks **E**), 6/3 (CtBP1/Pak1, **G**) muscles per group; **p < 0.01, ***p < 0.001, Student’s t-test

To get insights into the role of CtBP1 in muscle homeostasis, we first examined the expression pattern of CtBP1 isoforms in muscle cells. *Ctbp1-l* and *Ctbp1-s* transcripts, encoding CtBP1-L and CtBP1-S isoforms, arise from alternative splicing affecting the 5’ region and resulting in differential usage of ATG translation initiation codons and distinct exons 1 (Fig. S1A), while sharing all other exons (Fig. 1A). RT-PCR confirmed the expression of both *Ctbp1-l* and *Ctbp1-s* transcripts in C2C12 mouse myoblasts and myotubes (Fig. 1B). Using quantitative PCR (qPCR), their transcript levels were largely unchanged from myoblasts to 7-day-differentiated myotubes (Fig. 1C). RNAseq data available on *WashU Epigenome* Browser (15, 16) confirmed that levels of both transcripts are similar between undifferentiated and differentiated muscle cells in mice and humans (Fig. S1A). Notably, *CTBP1-L* levels, relative to *CTBP1-S* levels, were much lower in humans than in mice (Fig. S1A). At the protein level, CtBP1-S is 12 amino acids smaller than CtBP1-L, making the two isoforms indistinguishable by Western blot. Total CtBP1 protein levels were unchanged between C2C12 myoblasts and myotubes, while Pak1 expression progressively decreased upon muscle cell differentiation (Fig. S1B).

We next assessed the effect of neural activity on CtBP1 isoform expression pattern, by using sciatic nerve cut to trigger denervation of hind limb skeletal muscle. Transcript levels of *Ctbp1-l* and *Ctbp1-s* (Fig. 1D), as well as of total *Ctbp1* (*pan* – Fig. 1E), were unchanged in *tibialis anterior* (TA) 2 days after inducing denervation and slightly decreased in 3-week-denervated muscle compared to innervated muscle. Total CtBP1 protein levels were comparable in innervated and 16 h- to 3-week-denervated TA muscles (Fig. 1, F and G, Fig. S1C). In contrast, increase in *Pak1* transcript levels was detected in muscle 2 days after denervation (Fig. 1E) and the protein strongly accumulated after 7 days of denervation (Fig. 1, F and G, Fig. S1C), as previously reported [7]. CtBP1 levels remained unchanged after denervation in *soleus* muscle as well, while Pak1 accumulated as observed in TA muscle (Fig. S1D). Hence, although expression of the well-known CtBP1 regulator Pak1 depends on neural activity, accumulation of CtBP1 isoforms is unchanged upon denervation.

### CtBP1 accumulates in myonuclei shortly after denervation

**Fig. 2:**
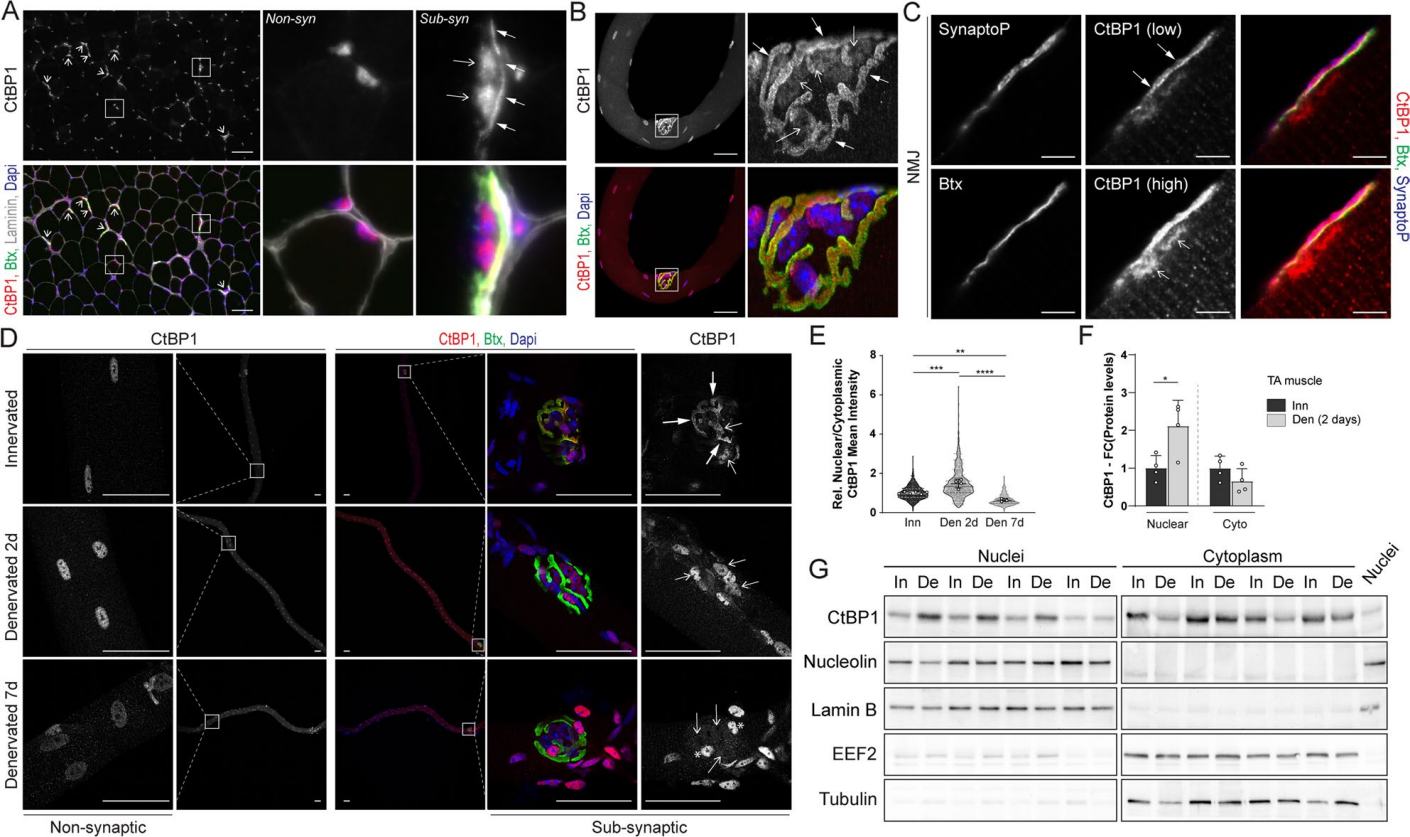
CtBP1 accumulates in myonuclei 2 days after denervation. **A** and **B** Staining of TA section (**A**) and EDL single fibers (**B**) with CtBP1 antibody and α-bungarotoxin (Btx) reveals accumulation of CtBP1 in non- and sub- (open arrows) synaptic (syn) myonuclei, as well as at the NMJ (arrows). Scale bar, 50µm. **C** STED microscopy of single fiber stained with CtBP1 and synaptophysin (SynaptoP) antibodies, as well as Btx, shows predominant CtBP1 accumulation in the NMJ pre-synaptic compartment. Low and high refer to low/high exposure time. Open arrows and arrows point to sub-synaptic nuclei and pre-synaptic CtBP1 staining, respectively. Scale bar, 5μm. **D** Immunostaining of innervated and denervated (2 and 7 days) EDL single fibers shows transient accumulation of CtBP1 in non- and sub- synaptic myonuclei, 2 days after denervation. CtBP1 pre-synaptic staining (arrows) is lost in denervated muscle fibers. Open arrows and asterisks point to sub-synaptic nuclei and non-muscle cells, respectively. Scale bar, 50μm. **E **Quantification of the ratio of mean CtBP1 fluorescence intensity in non-synaptic myonuclei of EDL isolated fibers, relative to cytoplasmic intensity and normalized to innervated muscle. Small dots represent individual nuclei. Large dots indicate the mean ratio per independent muscle (biological replicate), which was used for statistical analysis. **F** and **G** Levels of CtBP1 and sub-cellular markers in nuclear and cytoplasmic fractions of innervated (In) and 2-day-denervated (De) muscles. Quantification of CtBP1 levels in both fractions is given in F. Levels in nuclear and cytoplasmic fractions are normalized to levels of Lamin B and EEF2, respectively. All values are mean ± s.d.; n=6/4/3 (**E**) and 4 (**F**) independent muscles; *p<0.05, **p<0.01, ***p<0.001, ****p<0.0001; one-way ANOVA with Tukey’s post-hoc (**E**) and Student’s t-test (**F**)

A previous report links CtBP1 nuclear export with the release of synaptic gene repression in non-synaptic myonuclei 3 days after denervation [7]. To get further insights into CtBP1 function in activity-dependent processes, we first examined its sub-cellular distribution in sub- and non-synaptic muscle regions. Staining of transverse TA muscle sections with CtBP1 antibody and α-bungarotoxin (Btx), which binds AChRs and is used to visualize endplates, revealed a predominant accumulation of CtBP1 in sub- and non-synaptic nuclei. In addition, a non-nuclear CtBP1 signal was consistently detected at the NMJ (Fig. 2A). Immunostaining of isolated *extensor digitorum longus* (EDL) muscle fibers gave a faint cytoplasmic dotty pattern of CtBP1, together with a strong staining in myonuclei. CtBP1 was also strongly enriched at the NMJ, showing a similar pattern as Btx (Fig. 2B). *Ctbp1* knockdown using adeno-associated virus isotype 9 (AAV) carrying small hairpin RNA (shRNA) injected in TA/EDL muscles strongly reduced the staining observed, confirming its specificity (Fig. S2A). To further define the localization of CtBP1 at the NMJ, we used stimulated emission depletion (STED) microscopy with isolated muscle fibres. The non-nuclear CtBP1 staining at the NMJ co-localized predominantly with synaptophysin, a marker of the pre-synaptic compartment, adjacent to the Btx-labeled post-synaptic membrane, while only faint punctate staining was detected in the post-synaptic region (Fig. 2C). This pre-synaptic accumulation of CtBP1 is consistent with its known role in regulating synaptic vesicle dynamics in pre-synaptic active zones [18, 29]. We next examined temporal changes in CtBP1 localization induced by denervation, by analyzing TA muscle 16 h to 3 weeks after sciatic nerve cut. While CtBP1 distribution at 16 h and 7 days post-denervation was similar to innervated muscle, its nuclear accumulation increased 2 days and 3 weeks after denervation (Fig. S2B). These dynamic changes in CtBP1 localization were observed with two different antibodies (Fig. S2C). To go further, we stained single fibers isolated from denervated EDL muscle with CtBP1 antibodies and Btx. Consistent with muscle section immunostaining, CtBP1 staining increased in non- and sub-synaptic myonuclei after 2 days of denervation, compared to innervated fibers, with both antibodies (Fig. 2D and Fig. S2D). After 7 days of denervation, CtBP1 nuclear staining normalized and appeared more diffuse around myonuclei (Fig. 2D and Fig. S2E). Quantification of the mean fluorescence intensity of CtBP1 in non-synaptic nuclei confirmed its increased accumulation at 2 days of denervation, while it was reduced after 7 days (Fig. 2E). Of note, the non-nuclear CtBP1 staining at the NMJ, corresponding to the pre-synaptic compartment, was lost shortly after sciatic nerve cut (Fig. 2D), which may be caused by the release of CtBP1 from active zones upon nerve cut. To confirm the transient accumulation of CtBP1 in nuclei, we analyzed nuclear and cytoplasmic fractions of innervated and 2-day-denervated TA muscles. Consistent with the immunostaining results, CtBP1 levels were increased in nuclear fractions after 2 days of denervation, while remaining largely unchanged in cytoplasmic fractions (Fig. 2, F and G). Together these results indicate that denervation triggers dynamic changes in CtBP1 sub-cellular localization in both sub- and non-synaptic regions of muscle fibers (Fig. S2F). Interestingly, immunostaining of sections and isolated fibers showed that CtBP1 also accumulates in non- and sub-synaptic nuclei in *soleus* muscle (Fig. S2, G and H). However, its sub-cellular distribution remained largely unchanged 2 and 7 days after denervation, compared to innervated muscle (Fig. S2I). This result points to a fiber-type-specific regulation of CtBP1 trafficking, with denervation-induced relocalization occurring predominantly in fast, glycolytic muscles.

### *Ctbp1* knockdown has minor effect on histology of innervated and denervated muscles

**Fig. 3:**
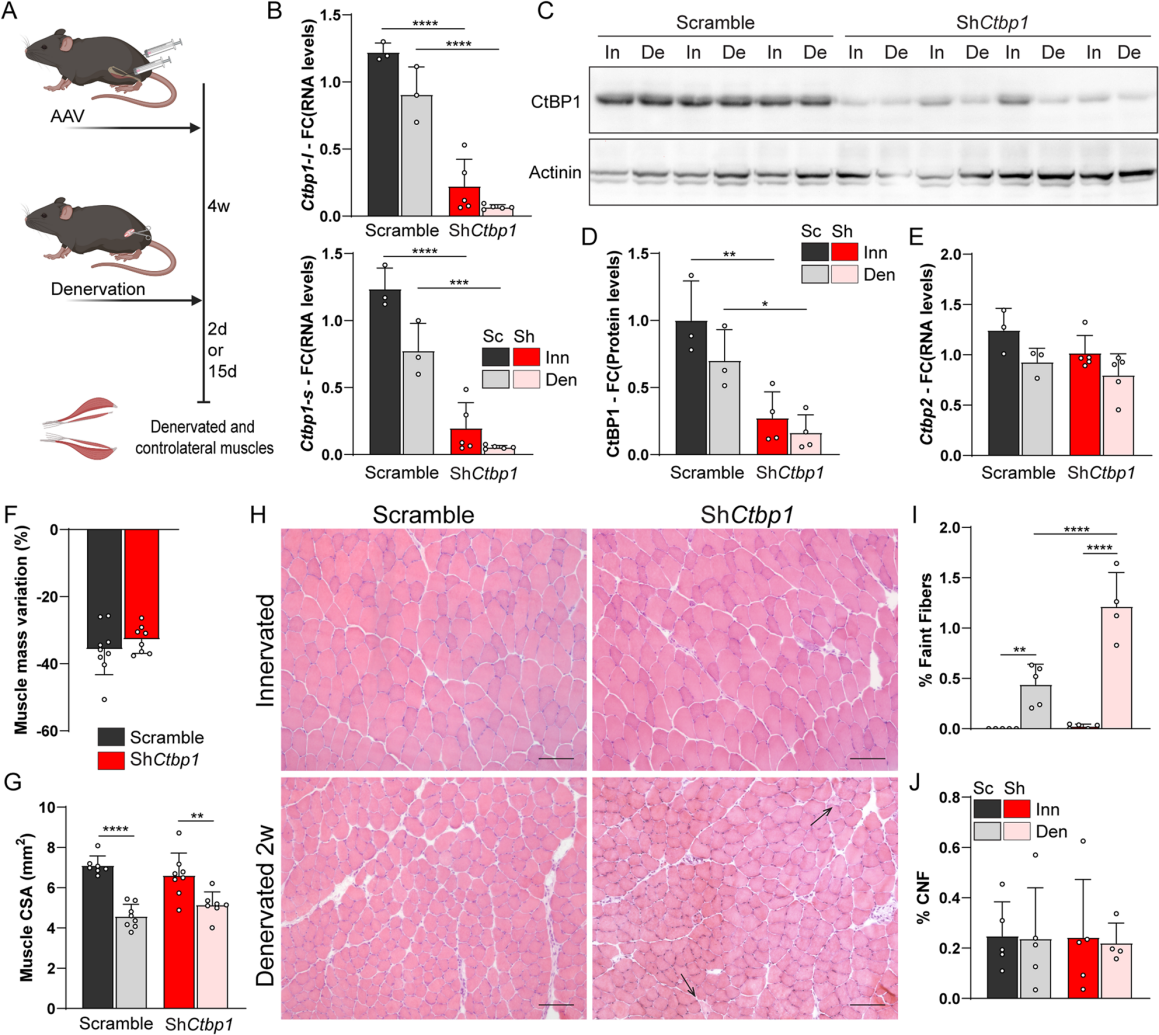
*Ctbp1 *knockdown has minor effect on muscle histology. **A** Sciatic nerve was cut unilaterally 4 weeks after injecting AAV-sh*Ctbp1* or -shScramble, and TA/EDL muscles were analyzed 2 or 15 days later. **B** mRNA levels of *Ctbp1-l* and *Ctbp1-*s in innervated (Inn) and denervated (Den, 2 days) TA muscles injected with AAV-sh*Ctbp1* (Sh) or -shScramble (Sc). Levels are relative to *Tbp* mRNA and to Scramble innervated. **C** and **D** Western Blot analysis of CtBP1 in innervated (In) and denervated (De, 2 days) TA muscles injected with AAV-sh*Ctbp1* or -shScramble. Protein levels are normalized to actinin, and relative to Scramble innervated. Quantification of CtBP1 levels is given in (**D**). **E** mRNA levels of *Ctbp2* in innervated (Inn) and denervated (Den, 2 days) TA muscles injected with AAV-sh*Ctbp1* (Sh) or -shScramble (Sc). Levels are relative to *Tbp* mRNA and to Scramble innervated. **F **Mass variation after 2 weeks of denervation for TA muscles injected with AAV-sh*Ctbp1* or -shScramble. **G** Cross sectional area (CSA) of innervated and denervated (2 weeks) TA muscles injected with AAV-sh*Ctbp1* or -shScramble. **H** H&E staining of innervated and 2-week-denervated TA muscles injected with AAV-sh*Ctbp1* or -shScramble. Arrows point to abnormally faint fibers. Scale bar, 100μm. **I** and **J** The proportion of abnormally faint fibers (**I**), but not of centronucleated fibers (CNF, **J**), is increased in denervated TA injected with AAV-sh*Ctbp1*, compared to Scramble. All values are mean ± s.d.; n=3Sc/5Sh (**B**, **E**), 3Sc/4Sh (**C**, **D**), 9Sc/8Sh (**F**), 7Sc/8Sh (**G**), 5 (**I** and **J**, except for Sh den, n=4) muscles per group; *p<0.05, **p<0.01, ***p<0.001, ****p<0.0001; two-way ANOVA with Tukey’s post-hoc

To assess the functional consequences of CtBP1 nuclear-cytoplasmic trafficking in denervated muscle, we injected AAV9-shRNA directed against *Ctbp1* in both hind limb anterior compartments of 3-month-old mice to knockdown *Ctbp1* expression in TA and EDL muscles. Four weeks later, sciatic nerve was cut unilaterally to compare the effect of *Ctbp1* knockdown in innervated and denervated muscles (Fig. 3A). A single injection of AAV-sh*Ctbp1* was sufficient to abolish *Ctbp1-l* and *Ctbp1-s* transcript levels (Fig. 3B) and to strongly reduce total CtBP1 protein levels in TA muscle, compared to control muscle injected with scramble shRNA (Fig. 3, C and D). Of note, CtBP1 pre-synaptic accumulation was preserved in innervated muscle fibers treated with AAV-sh*Ctbp1*, which was consistent with a predominant effect of the AAV9-shRNA in muscle fibers (Fig. S3A). As *Ctbp1* and *Ctbp2* transcripts share more than 70% sequence similarities, we evaluated *Ctbp2* expression by qPCR to assess the specificity of the AAV-shRNA and the lack of compensatory expression. Indeed, *Ctbp2* transcript levels were similar in TA muscles injected with AAV-sh*Ctbp1* or AAV-shScramble (Fig. 3E). Similarly, injection of AAV-shRNA directed against *Ctbp2* reduced specifically *Ctpb2* expression, while the co-injection of AAV-sh*Ctpb1* and AAV-sh*Ctpb2* decreased both *Ctbp1* and *Ctbp2* transcript levels (Fig. S3, B and C).

Two weeks after denervation, the loss of muscle mass and reduction in muscle cross sectional area were similar between mice injected with AAV-shScramble or AAV-sh*Ctbp1* (Fig. 3, F and G). AAV-sh*Ctbp1* injection did not alter muscle histology in innervated conditions. After 2 weeks of denervation, it slightly increased the proportion of abnormally faint fibers observed with H&E staining, which may correspond to pre-necrotic fibers (Fig. 3, H and I). However, the proportion of centronucleated fibers (i.e., regenerating) remained unchanged compared to control TA (Fig. 3J). There was also no major change in muscle mass loss or muscle histology after knocking down *Ctbp2* alone or both *Ctbp1* and *Ctbp2* (Fig. S3, D-H). Hence, these results indicate that reducing CtBP1 and/or CtBP2 levels has minor effect on muscle histology after denervation.

### *Ctbp1* knockdown does not perturb AChR dynamics and endplate maintenance

**Fig. 4:**
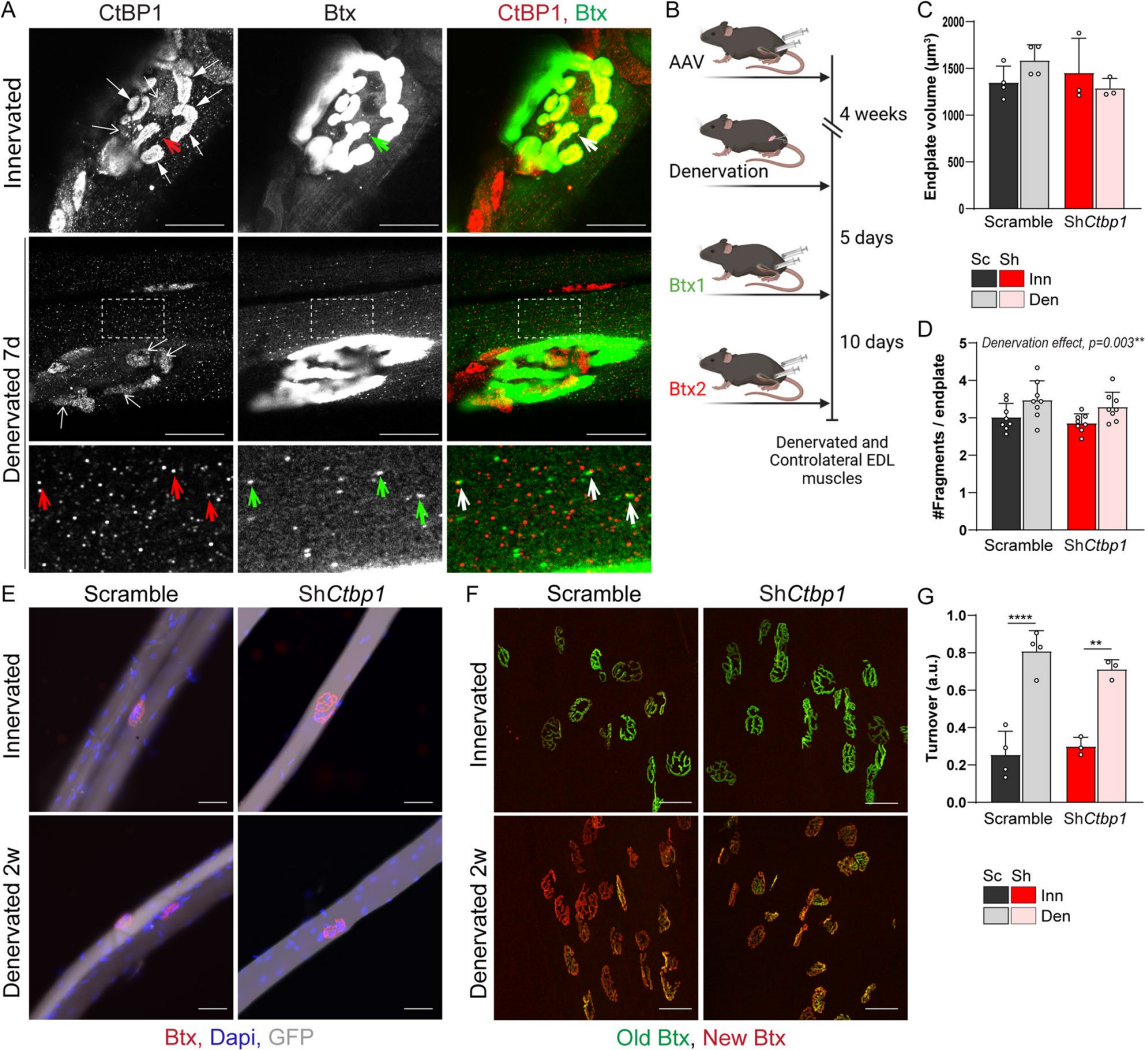
*Ctbp1* knockdown does not impair AChRs dynamics and endplate maintenance. **A** Immunostaining of permeabilized single EDL fibers with CtBP1 antibody and Btx shows that some AChR-containing vesicles stain positive for CtBP1 (green and red arrows) in innervated and denervated muscle. Arrows and open arrows point to CtBP1 staining in pre-synaptic compartment and sub-synaptic nuclei, respectively. Scale bar, 20µm.** B** Injection of AAV-sh*Ctbp1* and -shScramble was combined with Btx injection to analyze AChR turnover. **C**-**E** Btx staining of innervated (Inn) and denervated (Den) EDL fibers shows preserved endplate structure after AAV-sh*Ctbp1* (GFP-positive) infection (**E**). Quantification of endplate volume and fragmentation is given in **C** and **D**, respectively. Scale bar, 50µm. **F** and **G** Whole-mount Btx staining of innervated and 2-week-denervated EDL injected with AAV-sh*Ctbp1* or -shScramble distinguishes “old” (green) and “new” (red) AChRs. Quantification of AChR turnover is given in **G**. Scale bar, 50μm. All values are mean ± s.d.; n=8 (**D**), 4Sc/3Sh (**C**, **G**) muscles per group; **p<0.01, ****p<0.0001; two-way ANOVA with Tukey’s post-hoc

Previous reports establish that CtBP1 regulates synaptic vesicle exocytosis / recycling in pre-synaptic compartments [18, 29]. As we detected a faint dotty, non-nuclear staining for CtBP1 in the post-synaptic region of NMJ by STED, we examined whether it may correspond to vesicles enriched in newly formed or recycling AChRs. Consistent with increased AChR turnover upon denervation, Btx-positive dots in the endplate region, previously reported as endocytic vesicles containing AChRs [28, 30], accumulated further in single fibers isolated 7 days after denervation than in innervated fibers (Fig. 4A). Interestingly, some, but not all, Btx-positive vesicles co-localized with CtBP1 staining at the endplate (Fig. 4A). This suggests that CtBP1 may contribute to the regulation of post-synaptic vesicle trafficking. To assess the role of CtBP1 in AChR dynamics, we combined AAV-sh*Ctbp1* or AAV-shScramble injection with AChR turnover assay, using established procedures with sequential labelling of stable and newly formed AChRs with distinct fluorescent Btx (Fig. 4B) [28, 31]. AAV-sh*Ctbp1*-infected fibers from innervated and 2-week-denervated TA muscles did not show major endplate alteration, as compared to AAV-shScramble muscle (Fig. 4C-E). In particular, *Ctbp1* knockdown altered neither endplate volume (Fig. 4C) nor endplate fragmentation (Fig. 4D) in TA innervated and denervated muscles, as compared to control (shScramble) muscle. Consistent with AChR destabilization and increased formation of new receptors in muscle after nerve injury, AChR turnover was strongly increased in denervated muscles (Fig. 4, F and G). A similar increase in AChR turnover was observed after *Ctbp1* knockdown, suggesting that CtBP1 is dispensable for AChR dynamics (e.g., internalization and recycling) in innervated and denervated conditions. These results suggest that denervation-induced changes in CtBP1 nuclear-cytoplasmic trafficking are not coupled with sub-synaptic regulation of AChR-containing vesicles.

### CtBP1 regulates activity-dependent and -independent genes in skeletal muscle

**Fig. 5:**
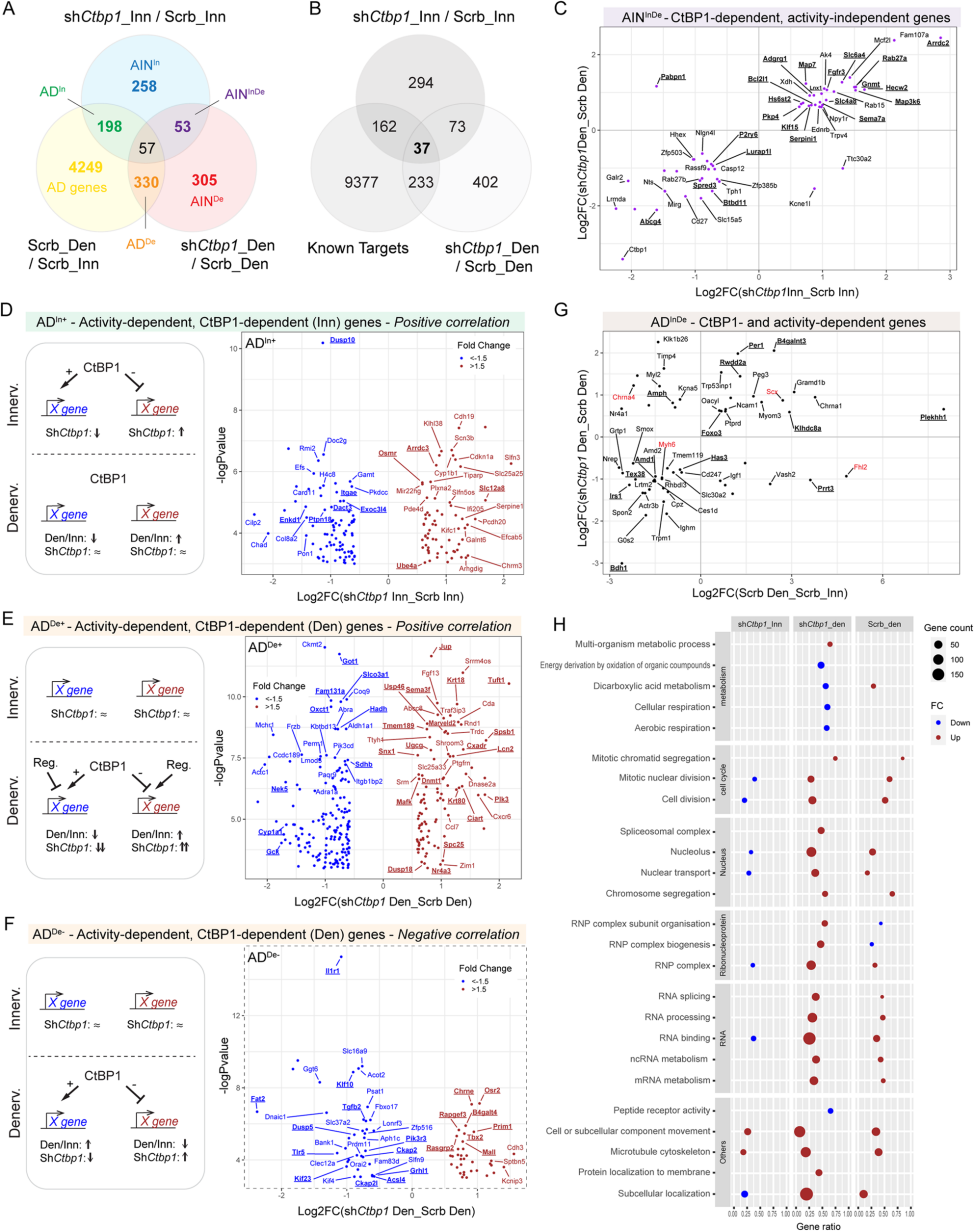
*Ctbp1* knockdown perturbs activity-dependent and -independent genes. **A** Venn diagram showing common differentially expressed (DE) genes between innervated (Inn) and 2-day-denervated (Den) muscles infected with AAV-shScramble (Scrb; yellow), innervated muscles infected with AAV-sh*Ctbp1* or -Scrb (blue), and 2-day-denervated muscles infected with AAV-sh*Ctbp1* or -Scrb (red). **B** Venn diagram showing common DE genes between CtBP1 known targets (extracted from TFlink.net), innervated muscles infected with AAV-sh*Ctbp1 *or -Scrb (dark grey), and denervated muscles infected with AAV-sh*Ctbp1* or -Scrb (white). **C** Scatter plot showing activity-independent genes DE in AAV-sh*Ctbp1* innervated muscles (vs. AAV-Scrb innervated) and in AAV-sh*Ctbp1* denervated muscles (vs. AAV-Scrb denervated). Only some genes show negative correlation between innervated and denervated changes. **D** *Ctbp1* knockdown has a similar effect on AD^In+^ genes in innervated muscle as denervation. The volcano plot shows genes DE in AAV-sh*Ctbp1* innervated muscles (vs. AAV-Scrb innervated), with positive correlation with denervation-induced changes. **E*** Ctbp1* knockdown exacerbates the denervation effect on AD^De+^ genes. The volcano plot shows genes DE in AAV-sh*Ctbp1* denervated muscles (vs. AAV-Scrb denervated), with positive correlation with denervation-induced changes. **F** *Ctbp1 *knockdown limits the denervation effect on AD^De-^ genes. The volcano plot shows genes DE in AAV-sh*Ctbp1* denervated muscles (vs. AAV-Scrb denervated), with negative correlation with denervation-induced changes. **G** Scatter plot showing activity-dependent genes DE in both AAV-sh*Ctbp1* innervated muscles (vs. AAV-Scrb innervated) and AAV-sh*Ctbp1* denervated muscles (vs. AAV-Scrb denervated). The plot shows the correlation between the effect of denervation and of AAV-sh*Ctbp1* in denervated muscle. Only some genes (red) show an inverse correlation in innervated muscle (see Fig. S4G). **H **GO enrichment analysis of genes DE after denervation and/or *Ctbp1* knockdown. Gene counts, up/down fold change, and gene ratio are indicated with the size, color and position of the bubble, respectively. n=3 muscles per group

CtBP1 transcriptional activity is associated with the repression of pro-apoptotic genes in cancer cells [32, 33], and of synaptic genes in innervated muscle [7]. To test whether the transient accumulation of CtBP1 in myonuclei of TA/EDL muscles 2 days after denervation is associated with the regulation of specific activity-dependent genes, we performed RNAseq analysis of innervated and 2-day-denervated TA muscles injected with either AAV-shScramble or AAV-sh*Ctbp1*. The expression pattern of denervated and innervated muscles strongly differs, irrespective of CtBP1 modulation (Fig. S4A). Comparison between innervated muscles injected with AAV-sh*Ctbp1* vs. -shScramble gave 566 differentially expressed genes (DEG; |FC|≥1.5, *p* < 0.05; Fig. 5A, blue circle), among which 53.0% were up-regulated with *Ctbp1* knockdown. Similarly, 745 DEG were identified in *Ctbp1*-silenced denervated muscle compared to control denervated muscle (|FC|≥1.5, *p* < 0.05; Fig. 5A, red circle), with 42.7% up-regulated genes. Interestingly, 48.7% of the genes deregulated with *Ctbp1* knockdown (in innervated and/or denervated muscles) were also differentially expressed between control innervated and denervated muscles (i.e., activity-dependent) (Fig. 5A). Notably, 36.0% corresponds to known CtBP1 target genes (Fig. 5B), based on publicly available transcription factor databases [34]. As recent evidence suggests that CtBP1 can also act as a transcriptional activator [35, 36], we considered genes up-regulated and down-regulated upon *Ctbp1* knockdown as direct or indirect targets repressed and induced by CtBP1, respectively.

Activity-independent DEG (hereafter referred to as AIN genes) included 53 genes deregulated in both innervated and denervated *Ctbp1*-silenced muscles (AIN^InDe^, Fig. 5, A and C). Other AIN genes were either deregulated in innervated (AIN^In^) or in denervated (AIN^De^) *Ctbp1*-silenced muscle compared to control (Fig. S4, B and C). Top AIN genes included *Ctbp1* as expected, as well as *Arrdc2*, *Sox7* and *Eppk1*, all three encoding factors involved in tumorigenesis and cell proliferation (Fig. 5C, Fig. S4, B and C).

Interestingly, among activity-dependent genes (AD), 198 were only affected by *Ctbp1* knockdown in innervated muscle (hereafter referred to AD^In^ genes, Fig. S4D). For most of them, their deregulation mimicked the denervation effect (positive correlation with sh*Ctbp1*, AD^In+^; Fig. 5D). Regulation of AD^In+^ genes would hence fit with the model proposed for synaptic genes [7], in which denervation-induced changes in gene expression relies on the release of the repressive effect exerted by CtBP1 in innervated muscle (Fig. 5D). In contrast, AD^In−^ genes (negative correlation with sh*Ctbp1*, Fig. S4E), such as *Fgfbp1*, would be regulated similarly by CtBP1 in innervated muscle and by another independent factor after denervation (Fig. S4E). In parallel, the deregulation of 330 activity-dependent genes only in denervated muscle (AD^De^) was consistent with CtBP1 accumulation in myonuclei 2 days after denervation (Fig. S4F). For most of these genes, the effect of denervation was dampened by CtBP1 activity (positive correlation with sh*Ctbp1*, AD^De+^; Fig. 5E). Top AD^De+^ genes included the metabolic genes *Gck*, *Ckmt2*, *Oxct1* and *Sdhb*, encoding glucokinase, mitochondrial creatine kinase, the ketolytic enzyme 3-oxoacid coA-transferase 1 and succinate dehydrogenase sub-unit B, respectively. These genes were down-regulated in denervated TA injected with AAV-shScramble, and even more with AAV-sh*Ctbp1*. Of note, *Tuft1*, which encodes a protein involved in oncogenesis and dental enamel mineralization [37] was the most up-regulated AD^De+^ gene. Its deregulation downstream of CtBP1 may play a critical role in HADTTS. Inversely, for some AD^De^ genes, the denervated effect may directly or indirectly rely on CtBP1 activity and its accumulation in nuclei (negative correlation with sh*Ctbp1*, AD^De−^; Fig. 5F). AD^De−^ genes included *Chrne* and *Tbx2*, encoding the adult ε sub-unit of AChR and TBX2, a known inhibitor of myogenesis [38]. Finally, 57 activity-dependent genes were DE in both innervated and denervated *Ctbp1*-silenced muscles compared to controls (referred to as AD^InDe^ genes; Fig. 5G). Only *Chrna4*,* Scx*,* Myh6* and *Fhl2* showed an inverse regulation by sh*Ctbp1* in innervated and denervated muscles (Fig. 5G and Fig. S4G). AD^InDe^ genes included *Foxo3*, a known target repressed by CtBP1 [39], and *Chrna1*, encoding AChR sub-unit α, which were both up-regulated by denervation and further induced in *Ctbp1*-silenced denervated muscle (Fig. 5G).

To get further insight into the overall effect on *Ctbp1* knockdown on the muscle response to denervation, we used gene ontology (GO) enrichment analysis comparing AAV-sh*Ctbp1 vs.* shScramble and innervated vs. denervated muscles. As expected with the oncogenic role of CtBP1, pathways associated with cell division and movement were deregulated in *Ctbp1*-silenced innervated muscle, compared to control muscle (Fig. 5H). Interestingly, *Ctbp1* knockdown led to a down-regulation of genes related to metabolic processes after denervation, as compared to control denervated muscle (Fig. 5H). In parallel, *Ctbp1* knockdown exacerbated the effect of denervation on the expression of genes related to cell cycle, nuclear regulation, RNA processing or (sub)cellular movement (Fig. 5H). Together, these results indicate that increased nuclear import of CtBP1 and its relocation on alternative target genes are required to release, limit or mediate the repression of specific synaptic and activity-dependent genes at early stages after denervation.

### CtBP1 regulates the expression of some AChR sub-units

**Fig. 6:**
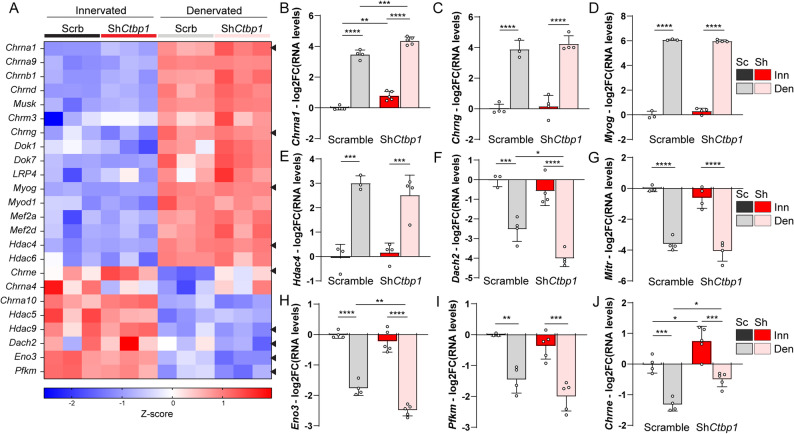
*Ctbp1* knockdown alters the activity-dependent expression of some synaptic genes. **A** Heatmap of z-scores computed based on log2 fold change of RNAseq counts for myogenic and synaptic genes in innervated and denervated, AAV-sh*Ctbp1* and -shScramble muscles. Expression of the genes pointed by an arrowhead was also quantified by qPCR. **B**-**J** mRNA levels of synaptic genes *Chrna1* (**B**), *Chrng* (**C**) and *Chrne *(**J**) and of the HDAC4 targets *Myog *(**D**),* Hdac4* (**E**), *Dach2* (**F**), *Mitr* (**G**), *Eno3* (**H**) and *Pfkm* (**I**), quantified by qPCR, after AAV-sh*Ctbp1* or -shScramble injection in innervated or 2-day-denervated TA muscles. Levels are relative to *Tbp* mRNA, normalized to AAV-shScramble innervated muscle, and analyzed as the log2 fold change (FC). Values are mean ± s.d.; n=4Sc/5Sh (**B**, **H**, **J**); 4ScInn/3ScDen/4Sh (**C**); 3Sc/4Sh (**D**, **E**); 3ScInn/4ScDen/4Sh (**F**, **G**); 3ScInn/4ScDen/5Sh (**I**) muscles per group; *p<0.05 **p<0.01 ***p<0.001 ****p<0.0001; two-way ANOVA with Tukey’s post-hoc

Up-regulation of synaptic genes after denervation relies on HDAC4 induction and the subsequent repression of *Dach2* and *Mitr/Hdac9*, which allows *Myog* expression (Fig. S5A) [3, 5, 8]. Release of CtBP1 repressive activity on *Myog* also contributes to *Myog* up-regulation upon denervation [7]. As synaptic processes did not appear in GO analysis, we examined individual RNAseq data for genes encoding synaptic proteins or proteins involved in their regulation (Fig. 6A). As expected, expression of most synaptic (e.g.,* Chrna1*,* Musk*) and myogenic (e.g.,* Myog*,* Mef2a*) genes, as well as *Hdac4*, increased in denervated muscle injected with AAV-shScramble (|FC|≥1.5, *p* < 0.05). Inversely, transcript levels of *Hdac9*, as well as *Eno3* and *Pfkm*, encoding metabolic enzymes, decreased 2 days after denervation (|FC|≥1.5, *p* < 0.05; Fig. 6A), as previously reported [10]. Notably, transcript levels of *Chrne*, encoding the adult ε sub-unit of AChRs, were reduced in control muscle at 2 days post-denervation (|FC|≥1.5, *p* < 0.05; Fig. 6A), while it was shown to be up-regulated at later stages [2]. Using qPCR, we confirmed that *Ctbp1* knockdown increases *Chrna1* levels in innervated muscle and further enhances its up-regulation in denervated muscle (Fig. 6B), as found with the RNAseq (AD^InDe^ gene; Figs. 5G and 6A). Levels of *Chrnb1* and *Chrnd* were also increased in *Ctbp1*-silenced denervated muscle, as compared to control muscle (|FC|≥1.25, *p* < 0.05; Fig. 6A). In contrast, levels of *Chrng*,* Myog* and *Hdac4* remained unchanged upon *Ctbp1* knockdown, by RNAseq (Fig. 6A) and qPCR (Fig. 6C-E). Knocking down *Ctbp2* or both *Ctbp1* and *Ctbp2* did not increase *Chrna1*, *Chrng* or *Myog* expression in innervated muscle (Fig. S5B). As CtBP1 further accumulated in myonuclei 2 days after denervation, we examined genes known to be repressed upon denervation. Unexpectedly, *Dach2*, but not *Mitr*, was further repressed upon denervation when knocking down *Ctbp1* (Fig. 6, F and G). *Ctbp1* knockdown also further repressed the expression of *Eno3*, but not of *Pfkm*, two genes targeted by HDAC4 [10] (Fig. 6, H and I). More interestingly, we identified that *Ctbp1* knockdown increases *Chrne* transcript levels in innervated muscle and dampened its repression upon denervation, in the RNAseq data (AD^De−^ with |FC|≥1.5, *p* < 0.05; Fig. 6A) and by qPCR (Fig. 6J). This indicates that CtBP1 represses *Chrne* in innervated muscle and contributes to its transient down-regulation 2 days after denervation, together with other repressors. These results indicate that temporal changes in CtBP1 localization contribute to the regulation of the expression of some synaptic genes, including those encoding AChR sub-units α1 and ε in innervated muscle and after denervation.

### CtBP1 restrains fiber type switch but does not interfere with denervation-induced atrophy

**Fig. 7:**
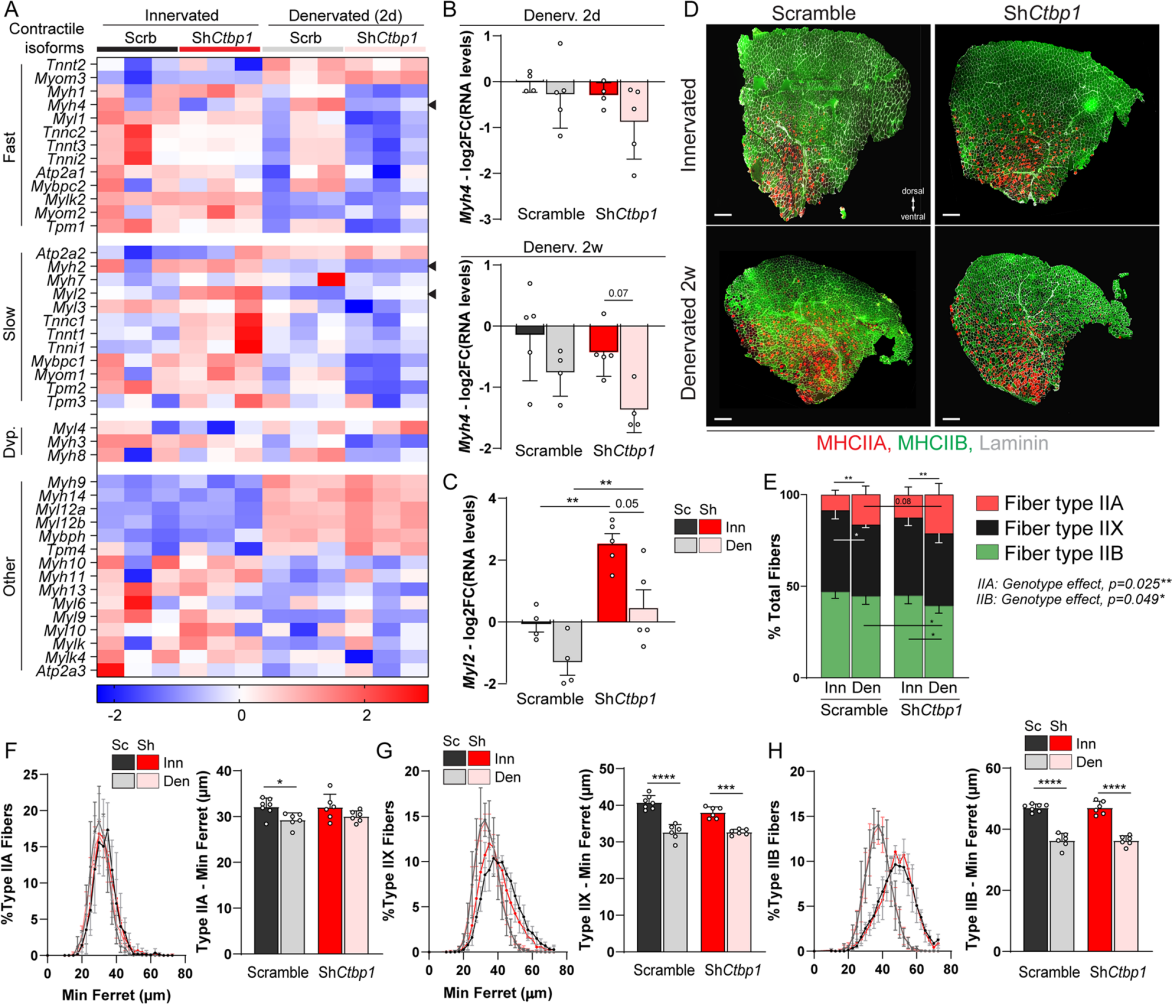
*Ctbp1* knockdown exacerbates the switch towards type IIA fibers.** A** Heatmap of z-scores computed based on log2 fold change of RNAseq counts for genes encoding fast, slow and developmental contractile protein isoforms in innervated and denervated, AAV-sh*Ctbp1 *and -shScramble muscles. Expression of the genes pointed by an arrowhead was also quantified by qPCR (see **B** and **C**, and Fig. S6A). **B** and **C** mRNA levels of* Myh4* (**B**, 2 and 15 days) and *Myl2 *(**C**, 2 days) after AAV-sh*Ctbp1* (Sh) or -shScramble (Sc) injection in innervated (Inn) or denervated (Den) TA muscles. Levels are relative to *Tbp* mRNA, normalized to AAV-shScramble innervated muscle, and analyzed as the log2 fold change (FC). **D** and **E** Immunostaining for type IIA and IIB myosin heavy chains (MHC) and laminin, of innervated and 2-week-denervated TA muscles infected with AAV-sh*Ctbp1* or -shScramble. The proportion of types IIA, IIX and IIB fibers in TA muscle sections is given in **E**. Scale bar, 500µm. **F-H** Fiber size distribution and mean minimum ferret for types IIA (**F**), IIX (**G**) and IIB (**H**) fibers in innervated (Inn) and 2-week-denervated (Den) muscles infected with AAV-sh*Ctbp1* (Sh) or -shScramble (Sc). All values are mean ± s.d.; n=4Inn/5Den (**B**, 2 days); 5Inn/4Den (**B**, 2 weeks); 4Sc/5Sh (**C**); 7ScInn/6ScDen/6ShInn/6ShDen (**E-H**) muscles per group; *p<0.05, **p<0.01, ***p<0.001, ****p<0.0001; two-way ANOVA with Tukey’s post-hoc

Denervation triggers severe muscle atrophy, mainly driven by HDAC4 and FoxO pathways [10, 40]. CtBPs were shown to repress FoxO3 activity and thereby to modulate the effect of the transcription factor ZEB1 on muscle atrophy [41]. In TA muscle, denervation-induced muscle atrophy affects predominantly type IIB and IIX fibers and is accompanied by a major shift towards type IIA oxidative fibers [10, 28]. We first used RNAseq data to evaluate changes in the expression of genes encoding slow, fast or developmental isoforms of contractile proteins after knocking down *Ctbp1*. Denervation had overall a mild effect on contractile genes 2 days after sciatic nerve cut (Fig. 7A). Interestingly, *Ctbp1* knockdown repressed genes encoding fast isoforms of some contractile proteins (e.g.,* Myh1* or *Myl1*) 2 days after denervation and increased the expression of genes encoding slow isoforms of myosin light chain (*Myl2*, slow/cardiac isoform) and troponin (*Tnnc1/t1/i1*) in innervated muscle (|FC|≥1.5, *p* < 0.05; Fig. 7A). RNAseq data (|FC|≥1.25, *p* = 0.055; Fig. 7A) and qPCR analysis (Fig. 7B) also showed that *Ctbp1* knockdown tends to repress *Myh4* (encoding myosin heavy chain (MHC) IIB) expression 2 days and 2 weeks after denervation. In parallel, *Myh2* (encoding MHCIIA) expression was strongly repressed 2 days after denervation, before being increased at 2 weeks (Fig. S6A), as previously reported [28]. However, *Ctbp1* knockdown did not alter *Myh2* transcript levels, compared to control muscle. In contrast, we confirmed by qPCR that *Ctbp1* knockdown strongly increases the expression of *Myl2* in innervated muscle and limits its repression 2 days after denervation (Fig. 7C).

As changes in contractile gene expression governs fiber type switch after nerve injury, we evaluated the proportion of each fiber type in innervated TA muscle and 2 weeks after denervation, based on immunostaining against MHCI/IIA/II. Type I fibers were rarely detected in TA muscles across all experimental conditions and were mostly intrafusal (Fig. S6B). At 2 weeks, denervation increased the proportion of type IIA fibers in TA muscle, while the proportion of type IIX fibers was reduced (Fig. 7, D and E). *Ctbp1* knockdown increased the proportion of type IIA fibers and reduced the proportion of type IIB fibers, as compared to control muscles, resembling the fiber-type shift induced by denervation (Fig. 7, D and E). These results indicate that CtBP1 sustains the innervated pattern and restrains the effect of denervation on fiber type shift, likely by regulating the transcriptional levels of contractile proteins.

We next evaluated the effect of knocking down *Ctbp1* on muscle atrophy. RNAseq data confirmed that genes encoding proteins involved in catabolic processes, i.e., proteasome machinery, calpains, autophagic components, were strongly up-regulated 2 days after denervation (Fig. S6C). However, their expression was largely unchanged between control and *Ctbp1*-silenced muscles, including for the atrogenes *Fbxo32* and *Trim63*, which are directly regulated by FoxO3. Only *Fbxo40* and *Nedd4*, which encode two E3 ubiquitin ligases associated with denervation-induced muscle atrophy [42, 43], showed reduced levels with *Ctbp1* knockdown in denervated muscle (Fig. S6C). By qPCR, we confirmed that *Nedd4* expression is repressed in innervated muscle injected with AAV-sh*Ctbp1* and showed that its up-regulation after 2 weeks of denervation is limited in *Ctbp1*-silenced muscle, as compared to control muscle (Fig. S6D). In contrast, there was no major change in *Fbxo40* levels between mutant and control muscle 2 weeks after denervation (Fig. S6D). These results indicate that *Ctbp1* knockdown has minor effect on atrophy-related gene expression in innervated muscle and after denervation. Consistently, *Ctbp1* knockdown did not alter fiber size, irrespective of the fiber type, in innervated muscle (Fig. 7F-H). Furthermore, it did not prevent or exacerbate the reduction in fiber size induced by denervation (Fig. 7F-H). These results demonstrate that CtBP1 restrains the contractile fiber type switch induced by denervation, without interfering with atrophying processes.

### CtBP1 limits the shift to oxidative fibers and mitochondrial fragmentation

**Fig. 8:**
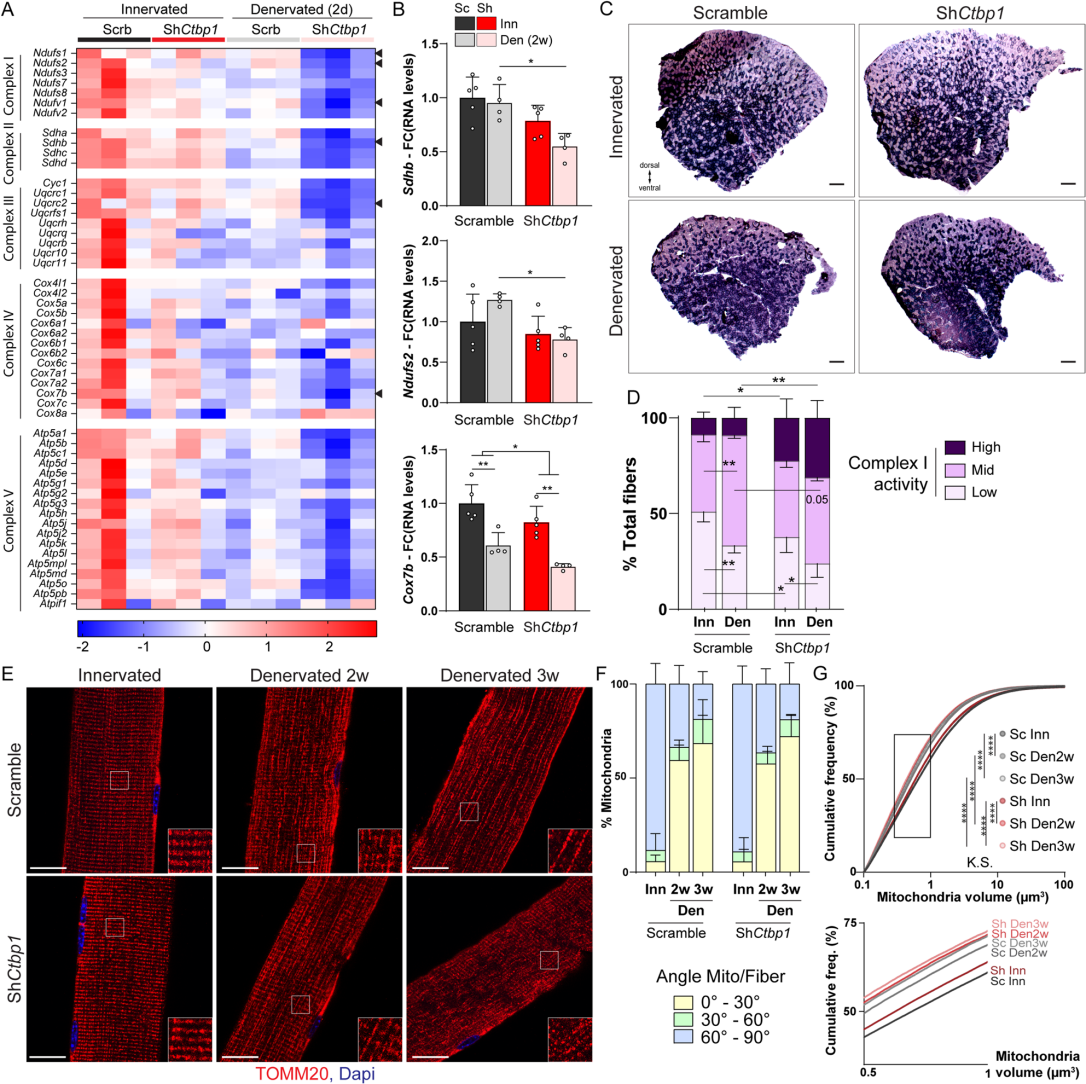
*Ctbp1 *knockdown alters mitochondria activity and remodeling. **A** Heatmap of z-scores computed based on log2FC of RNAseq counts for nuclear genes encoding proteins of respiratory chain complexes I to V, in innervated and 2-day-denervated, AAV-sh*Ctbp1* and -shScramble muscles. Expression of the genes pointed by an arrowhead was also quantified by qPCR at 2 weeks of denervation (see **B** and Fig. S8A). **B** mRNA levels of genes encoding components of the respiratory complex I (*Ndufs2*), II (*Sdhb*) and IV (*Cox7b*) after AAV-sh*Ctbp1* (Sh) or -shScramble (Sc) injection in innervated (Inn) and 2-week-denervated (Den) TA muscles. Levels are relative to* Tbp* mRNA and normalized to AAV-shScramble innervated muscle. **C** and **D** NADH staining of innervated and 2-week-denervated TA muscles injected with AAV-sh*Ctbp*1 or -shScramble. The proportion of fibers with high-, mid- and low-intensity staining is given in **D**. Scale bar, 500μm. **E-G **TOMM20 immunostaining of innervated and 2-/3-week-denervated EDL muscles injected with AAV-sh*Ctbp1* or -shScramble shows mitochondria reorientation in denervated muscles. The proportion of mitochondria showing an angle of 0-30°, 30-60° or 60-90° as compared to the fiber is given in **F**. The cumulative frequency of mitochondria according to their volume is given in **G**. The 3D reconstitution of the pictures is given in Fig. S9D. Scale bar, 20µm. All values are mean ± s.d.; n=5Inn/4Den (**B-D**), 5 **(E-G**) muscles per group; *p<0.05, **p<0.01, ****p<0.0001; two-way ANOVA with Tukey’s post-hoc (**B, D, F**), Kolmogorov-Smirnov’s test (**G**)

GO analysis suggests a major role of CtBP1 in regulating metabolic paths, including cell respiration, in denervated muscle (Fig. 5H). Although denervation induces a switch from glycolytic to oxidative fibers in TA muscle, its effect on the different metabolic processes remains unclear. We showed that denervation perturbs the expression of genes associated with glycolysis (Fig. S7A) and tricarboxylic acid cycle (TCA; Fig. S7B), although with a mixed effect, with genes up- and down-regulated in each path in 2-day-denervated muscle. In contrast, nuclear genes encoding sub-units of the respiratory chain (complex I to V) were overall repressed 2 days after denervation (Fig. 8A and Fig. S7C). Importantly, repression of these metabolic genes was enhanced by *Ctbp1* knockdown (AD^Den+^ genes; Fig. 8A and Fig. S7A-C), indicating that CtBP1 antagonizes the metabolic effect of denervation. Two weeks after denervation, expression of *Ndufs1*,* Ndufs2*,* Ndufv1* and *Sdhb* (encoding proteins of the respiratory chain) normalized to innervated muscle (Fig. 8B and Fig. S8A). Only *Cox7b* levels remained lower in 2-week-denervated muscle (Fig. 8B). At this stage, the repressive effect of AAV-sh*Ctbp1* on these metabolic genes was still detected, as compared to control (shScramble) denervated muscle (Fig. 8B and Fig. S8A). However, despite these transcriptional changes, protein levels of complex I to V of the respiratory chain remained similar after denervation or *Ctbp1* knockdown, 2 days and 2 weeks after denervation (Fig. S8, B-E). This discrepancy may involve low turnover rate for complexed proteins of the respiratory chain.

To test whether CtBP1 deficiency has an impact on respiratory chain function, we assessed complex I activity by performing NADH staining on TA muscle sections (Fig. 8C). As expected with the oxidative shift of TA muscle after nerve cut, the proportion of fibers with low NADH staining decreased 2 weeks after denervation in control muscle (Fig. 8D). Interestingly, *Ctbp1* knockdown exacerbated this switch, with a reduced proportion of fibers with low NADH staining in innervated muscle, and an increased proportion of fibers with high NADH staining in both innervated and denervated muscles, compared to controls (Fig. 8D). This indicates that reducing CtBP1 levels increases complex I activity and precipitates the oxidative switch occurring after denervation. Of note, 2-week denervation and *Ctbp1* knockdown had limited effect on the activity of complexes II (SDH staining; Fig. S8, F and G) and IV (COX staining; Fig. S8, H and I).

To go further, we examined mitochondria network in innervated and denervated muscles using electron microscopy (EM). Using longitudinal sections, we identified that mitochondria size increases in control muscle 2 weeks after denervation, leading to elongated structures in between sarcomeres (Fig. S9A). Consistently, mitochondria area increased, while their mean circularity decreased after denervation (Fig. S9, B and C). Interestingly, *Ctbp1* knockdown dampened denervation-induced changes in mitochondria size, leading to reduced mitochondria area in denervated muscle, as compared to control denervated muscle (Fig. S9, A-C). To further characterize mitochondria remodeling upon denervation, we immunostained isolated fibers for TOMM20, a marker of the outer mitochondrial membrane (Fig. 8E). Using 3D analysis, we uncovered that the mitochondria network shifts from a transversal orientation compared to fibers, to a longitudinal orientation after 2 and even more so 3 weeks of denervation (Fig. 8E and Fig. S9D). Using MATLAB software, we showed that the proportion of mitochondria forming an angle of 60–90° with the fiber decreased from 88.5% to 18.7% 3 weeks after denervation (Fig. 8F). This result indicates that the apparent increase in mitochondria surface observed in longitudinal muscle sections by EM reflects a change in orientation of the network after denervation. This shift was also observed in muscle injected with AAV-sh*Ctpb1*, indicating that the reduced mitochondria area detected by EM does not correspond to a defective re-orientation of the network. We thus evaluated changes in mitochondria volume using Imaris 3D volumetric reconstitution (Fig. S9D). In both control and *Ctbp1*-silenced muscles, the median mitochondria volume decreased after 2 and 3 weeks of denervation, compared to innervated muscle (Fig. S9E). Consistently, the frequency distribution of mitochondria volumes was shifted to smaller mitochondria in control (shScramble) muscle after 2 and 3 weeks of denervation (Fig. 8G). Interestingly, *Ctbp1* knockdown already shifted the frequency distribution to smaller mitochondria in innervated muscle, and the distribution at 2 weeks of denervation was similar to the distribution observed at 3 weeks of denervation in control muscle (Fig. 8G). Hence, reducing CtBP1 levels mimics denervation-induced mitochondria size reduction in innervated muscle, and amplifies mitochondria fragmentation in denervated muscle, without altering the re-orientation of the network. Of note, *Ctpb1* overexpression or knockdown in C2C12 myoblasts did not perturb the morphology of the mitochondria network suggesting that the effect of CtBP1 on mitochondria is limited to mature muscle fibers (Fig. S9, F-J). Moreover, mitochondria remodeling after denervation and *Ctbp1* knockdown occurred without any changes in the transcript levels of genes encoding mitochondria fusion/fission machinery (e.g.,* Mfn1/2*, *Opa1*,* Bax/Dnm1l*) 2 days (Fig. S10A) and 2 weeks (Fig. S10B) after sciatic nerve cut. There was also no change in OPA1 and MFN1 protein levels in denervated and/or *Ctbp1*-silenced muscles (Fig. S10, C-F). Although levels of active and inactive phosphorylated forms of DRP1 were altered after denervation, there was no major difference between control and *Ctbp1*-silenced muscles (Fig. S10, C-F). Similarly, protein levels of PINK1, involved in mitophagy, were reduced 2 weeks after denervation; however, no change was detected after knocking down *Ctbp1* (Fig. S10, E and F). These results suggest that CtBP1 may regulate mitochondria fusion/fission and/or mitophagy in mature muscle fibers, without interfering with the expression of key regulatory genes. Together, these data demonstrate that CtBP1 sustains metabolic properties of skeletal muscle fibers, by limiting metabolic gene repression and mitochondria fragmentation.

## Discussion

Denervation triggers major synaptic, metabolic and contractile changes in muscle fibers, which are tightly regulated with time. Although different factors have been shown to contribute to this complex remodeling of denervated fibers, the integrated mechanisms underlying the muscle response to nerve injury are not fully understood. Here, we establish that CtBP1 activity, likely linked to its dynamic nuclear-cytoplasmic trafficking, is essential for the regulation of synaptic and metabolic gene expression and that CtBP1 prevents excessive metabolic and contractile shift after denervation.

The vast majority of CtBP1 studies concerns its oncogenic role via transcriptional regulation of apoptosis, inflammation and cell cycle [22]. In the neuromuscular system, CtBP1 is linked to the repression of myogenic and synaptic genes in mature muscle fibers [7], as well as with pre-synaptic vesicle recycling [18, 29]. We here showed that CtBP1 is present in both sub- and non-synaptic myonuclei. Moreover, its nuclear accumulation transiently increased 2 days after denervation in both types of myonuclei in TA/EDL muscles, independently from changes in its protein levels. This temporal regulation suggests that CtBP1 exerts activity-dependent functions in myonuclei and/or cytoplasm. Protein levels of Pak1, which drives CtBP1 nuclear export 3 days after denervation [7], were not yet changed 2 days after denervation. Different post-translational modifications regulate CtBP1 nuclear-cytoplasmic trafficking in non-muscle cells. Especially, small ubiquitin-like modifier (SUMO) conjugation (sumoylation) of CtBP1, involving PIAS, RanBP2 or Cbx4 E3 ubiquitin ligases, increases its nuclear import [44–46]. HDAC4 nuclear import, which also increases shortly after denervation, is promoted as well by RanBP2-dependent sumoylation [47]. Inversely, activity of the myogenic transcription factors MEF2, which is tightly regulated after denervation, is inhibited by sumoylation [48, 49]. Whether sumoylation changes contribute to CtPB1 nuclear-cytoplasmic trafficking in skeletal muscle requires further investigation. In parallel, the modest decrease in *Ctbp1* transcripts observed after prolonged denervation may reflect transcriptional reprogramming secondary to atrophy and metabolic changes, with the potential involvement of known (e.g., NRF1 [50]) or predicted (e.g., NFATc1 [51]) *Ctbp1* regulators that are perturbed after denervation.

The nuclear accumulation of CtBP1 2 days after denervation in TA/EDL muscles raised the question on the genes targeted at this stage. Knockdown assay confirmed that CtBP1 contributes to the repression of *Chrna1* in innervated muscle, while minor effect was found for *Myog* and *Chrng*. The use of AAV prevented muscle necrosis, which could account for the observed discrepancy in *Myog* expression changes compared to the results reported by Thomas et al., who employed muscle electroporation [7]. Up-regulation of *Chrna1*,* Chrnb1* and *Chrnd* was also enhanced after denervation in *Ctbp1*-silenced muscle, while *Myog* expression was similarly increased. By forming repressive complex with MITR, HDAC7 and/or ZEB1, CtBP1 may directly inhibit MEF2 factors at E-boxes of some synaptic gene promoters and thereby repress their expression in non-synaptic muscle regions, independently from myogenin regulation [52–54]. We further establish that CtBP1 plays a critical role in the regulation of *Chrne* expression. The selective expression of *Chrne* in sub-synaptic myonuclei, even after denervation, is linked to permissive effectors, such as p300 and ETV5/GABP, acting at the NMJ [55, 56]. We identified that *Chrne* expression decreases quickly after denervation, before increasing as described before [2]. *Ctbp1* knockdown increased *Chrne* expression in innervated muscle and limited its repression after denervation. CtBP1 may hence directly inhibit *Chrne* expression by forming repressive complex with HDAC1 and/or inhibiting p300 activity at ETS-binding sites of *Chrne* promoter in non- and sub-synaptic regions, respectively [55, 57, 58]. Especially, *Chrne* repression may rely on CtBP1 transient import in sub-synaptic myonuclei shortly after denervation. Other repressors may contribute to *Chrne* repression at this stage, as *Ctbp1* knockdown did not abolish the repressive effect of denervation on the gene.

In neurons, CtBP1 traffics between nuclei and pre-synaptic compartments, where it regulates genes involved in synaptogenesis and synaptic vesicle recycling, respectively [18, 29]. Its localization is tightly regulated by neural activity and relies on CtBP1interaction with Bassoon/Piccolo in active zones. At the NMJ, we identified that pre-synaptic CtBP1 is lost quickly after axonal injury. Sciatic nerve cut may provoke massive release of pre-synaptic vesicles and local CtBP1 degradation during Wallerian degeneration. By analogy with neurons, we hypothesized that CtBP1 nuclear export detected 7 days after denervation may associate with synaptic functions. CtBP1 was present in some internalized AChR-containing vesicles at the endplate. However, *Ctbp1* knockdown did not affect endplate maintenance or AChR turnover in innervated and denervated muscles. This suggests that CtBP1 is dispensable for the regulation of post-synaptic vesicle recycling and trafficking. 

In parallel with synaptic remodeling, we uncover that CtBP1 plays a key role in the regulation of metabolic and contractile properties of skeletal muscle after denervation. *Ctbp1* knockdown exacerbated the repression of genes related to glycolysis and mitochondrial respiration at 2 days of denervation. This result indicates that CtBP1 nuclear import is required to restrain metabolic gene repression upon denervation. CtBP1 may act as a transcriptional activator on these genes, as described in some inflammatory responses when complexed with p300 [35, 36], or it may indirectly promote their expression by repressing the expression or inhibiting the activity of specific transcriptional repressors. In several cell types, CtBP1 acts as a metabolic sensor by binding NADH, and to a lower extent NAD^+^ [59–61]. NADH binding regulates the nuclear import, the oligomerization and the transcriptional activity of CtBP1 [62–66]. Interactions of CtBP1 with transcriptional co-regulators are either increased or released upon NADH binding, leading to distinct transcriptional consequences upon changes in NADH/NAD^+^ levels [66–68]. Interestingly, denervation confers transient insulin resistance to skeletal muscle after 1–3 days [69, 70]. This complex effect may involve changes in Akt activity, as well as opposite changes in *Glut1/Glut4* expression [71–73]. By perturbing glycolytic flux and thereby free NADH levels, insulin resistance may trigger changes in CtBP1 activity shortly after denervation. Especially, this may release CtBP1 activity on some genes 2 days after denervation (e.g.,* Chrna1*), while reinforcing its repressing or permissive activity on others (e.g., metabolic genes). Despite its repressive effect on metabolic genes, *Ctbp1* knockdown reproduced in innervated TA muscle, the transition to slower, oxidative fibers triggered by denervation, and exacerbated contractile and metabolic changes in denervated muscle. The switch towards slower oxidative fibers in fast TA muscle after denervation involves the combined induction of HDAC4 [10], PGC1α [74] and myogenin [75]. In this context, changes in mitochondria network have been debated [50, 76–82]. We here report that denervation triggers profound mitochondria remodeling with a re-orientation of the network and a reduction in mitochondria volume. These changes likely involve finely tuned regulation of mitochondria fusion/fission and mitophagy. The mitochondria network reorientation may reflect the switch to oxidative fibers in TA muscle and/or the reinduction of the embryonic program after denervation [83]. In line with this, *Ctbp1* knockdown precipitated the oxidative switch of TA muscle and the reduction in mitochondria volume induced by denervation. The expression of *Bax*, previously shown to link CtBP1 with mitochondria fusion [32], remained unchanged in *Ctbp1*-silenced skeletal muscle. As for Golgi and vesicle membrane fission, CtBP1 may promote mitochondria fusion, by modulating lipid organization and/or components of the fusion/fission machinery. Importantly, two reports suggest that mutations in *CTBP1* gene cause mitochondria dysfunction with reduced complex I and IV activity in muscle from HADDTS patients [20, 21, 32]. The two corresponding mutations (p.Arg331Trp and p.Gln439ValfsTer84) are in the PXDLS domain of CtBP1 and may alter its interactions with DNA-binding proteins. Abnormal repression of metabolic genes and mitochondria fragmentation, as observed with *Ctbp1* knockdown, may contribute to mitochondria dysfunction in patients. Muscle biopsies from HADDTS patients also display predominance of slow fiber types, which was consistent with the exacerbated switch towards type IIA fibers in *Ctbp1*-silenced TA muscle. Accordingly, we showed that CtBP1 regulates some genes encoding fast and slow contractile protein isoforms. Finally, despite the reported role of CtBP1 in repressing FoxO3 activity [39] and the increased expression of *Foxo3* detected by RNAseq after *Ctbp1* knockdown, reducing CtBP1 levels did not perturb denervation-induced muscle atrophy and had no major effect on atrophy-related gene expression.

## Conclusions

In conclusion, our work identifies an important role of CtBP1 in maintaining activity-dependent properties of skeletal muscle fibers and dampening contractile, metabolic and synaptic remodeling induced by denervation. CtBP1 deregulation may hence contribute to the complex pathogenic effects leading to muscle dysfunction in neuromuscular diseases and sarcopenia.

## Supplementary Information

Supplementary Material 1.

## Data Availability

All data needed to evaluate the conclusions in the paper are present in the paper and Supplementary Materials. Data generated during the study are freely accessible on the Yareta repository database (10.26037/yareta:t5p7zkd7z5btrid6ijkz6rgx2u); RNAseq data will be deposited in Gene Expression Omnibus (GEO).

## Abbreviations

AAV: Adeno-Associated Virus
AChR: Acetylcholine Receptors
AIN: Activity-Independent gene
AD: Activity-Dependent gene
Btx: α-bungarotoxin
DEG: Differentially Expressed Gene
EDL: Extensor digitorum longus
EM: Electron Microscopy
FC: Fold Change
GO: Gene Ontology
HADDTS: Hypotonia, Ataxia, Developmental Delay and Tooth-enamel syndrome
NADH-TR: NADH tetrazolium reductase
NMJ: NeuroMuscular Junction
PFA: ParaFormAldehyde
shRNA: Small hairpin RNA
STED: STimulated Emission Depletion microscopy
TA: Tibialis anterior
