# Supplementary material for: CtBP1 sustains activity-dependent muscle properties and dampens synaptic, contractile and metabolic changes triggered by denervation
Source: Skelet Muscle. 2026 Mar 23;16:19. doi: 10.1186/s13395-026-00421-w (PMC13130457; doi:10.1186/s13395-026-00421-w)
Supplement: Supplementary file 1 — Supplementary Material 1. [file 13395_2026_421_MOESM1_ESM.pdf]

Supplementary Materials

CtBP1 sustains activity-dependent muscle properties and dampens synaptic, contractile and metabolic changes triggered by denervation

Olivia Cattaneo, Gaetan Lopez, Jayasimman Rajendran, Florent Chabry, Nicolas Liaudet, Sergei Startchik, Alexandre Prola, Perrine Castets

Supplementary Materials include: supplementary methods, 10 supplementary figures, 1 supplementary table, supplementary references.

Supplementary Methods

In vitro mitochondria analysis

C2C12 myoblasts were grown on 8 well chamber slides (80826, Ibidi) and transfected with overexpressing plasmids (CtPB1, MC215778 from Origene; RFP, pCMV-tdRFP) using JetOPTIMUS reagent (Polyplus), or alternatively with siRNA (Silencer Pre-Designed siRNA against *Ctbp1*, AM16708 and Silencer Cy3-labeled Negative Control siRNA, AM4621 from ThermoFisher), using Lipofectamine RNAiMAX (ThermoFisher), as previously reported (1). 24h or 48h after transfection, cells were incubated for 30 min at 37°C with prewarmed growth medium containing 200 nM MitoTracker Green FM probe (ThermoFisher), put back in growth medium, and analyzed with time lapse videos using a Nikon A1R spectral confocal microscope. Mitochondria were evaluated every 10 sec during 2 min in transfected cells, with ImageXpress software (Molecular Devices, Sunnyvale, CA; version 6.7.2.290) using the tools Angiogenesis Tube Formation and Integrated Morphometry Analysis to detect and analyze mitochondria segments, their junctions and length.

Electron microscopy

EDL muscles were pinned on a gel coated petri dish and fixed with 2.5% glutaraldehyde in 0.1M phosphate buffer, pH 7.2, at 4°C for one hour. Muscles were post-fixed in a tube with 2.5% glutaraldehyde in 0.1M phosphate buffer, pH 7.2, at 4°C for one hour. Muscles were then washed with phosphate buffer and further treated with 2% osmium tetraoxyde in buffer and immersed in a solution of uranyl acetate 0.25% over night to enhance contrast of membranes. Small pieces of EDL muscles were gradually dehydrated in ethanol (30% to 100%) and substituted gradually in a mix of propylene oxyde-epon and embedded in Epon (Delta microscopy). Thin sections (70 nm) were collected onto 200 mesh copper grids, and counterstained with uranyl acetate and lead citrate. Grids were examined with a Morgagni electron microscope (FEI Company, Eindhoven, Netherlands) operating at 80 kV. Images were acquired with a charge-coupled device camera (AMT).

Images analysis

Images were analyzed with Zen lite, LAS X, Fiji, Imaris and MATLAB software. 3D confocal images of TOMM20 were analyzed with Imaris 10.2.0 using the Surfaces model generated through software's machine learning-based segmentation methods. The volume of individual mitochondria was quantified. COX and SDH staining were analyzed using QuPath v0.6.0, with individual fiber segmentation using Cellpose-SAM. Staining intensity was quantified as the mean optical density summed across the red, green, and blue channels. To account for inter-image staining variability, intensities were normalized using linear scaling based on the 5th and 95th percentiles of the fiber intensity distribution and each fiber's intensity was rescaled relative to this interval.

Supplementary Figures

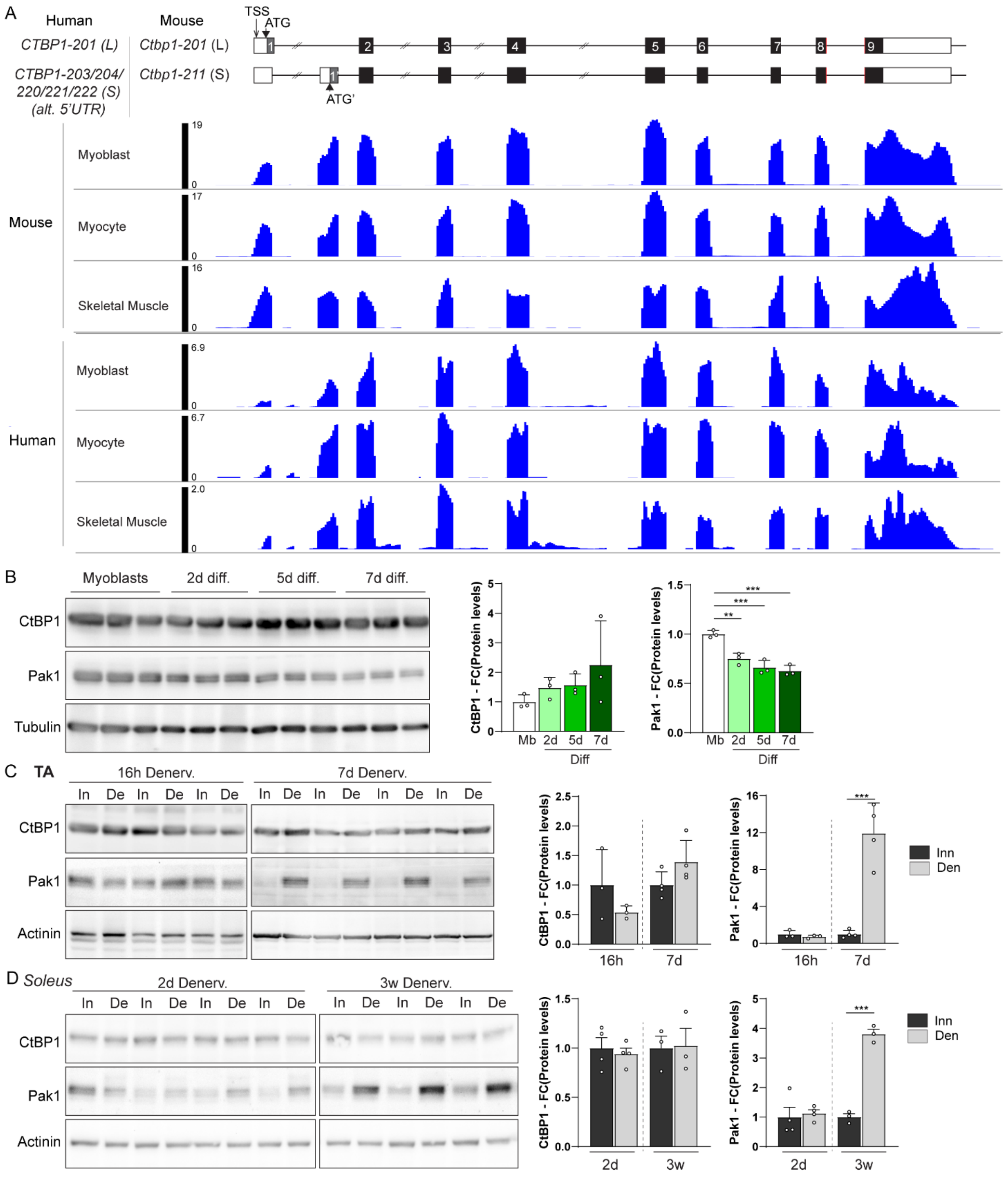

Fig. S1: Expression of CtBP1 isoforms is unchanged upon C2C12 differentiation and muscle denervation. **(A)** Organization of *Ctbp1/CTBP1* genes in mouse and human. Exons, introns and untranslated (UTR) sequences are represented by grey boxes, black lines and white boxes, respectively. Alternative ATG codons are indicated with arrows. RNAseq data from *WashU Epigenome Browser* (2, 3) confirm the expression of *Ctbp1-l* and *Ctbp1-s* in muscle cells from mouse, while human muscle

cells express mainly *CTBP1-S*. (**B**) Western Blot analysis of CtBP1 and Pak1 in C2C12 myoblasts and after 2 to 7 days (d) of differentiation (diff). Protein levels are normalized to tubulin and to myoblasts. (**C**) Western Blot analysis of CtBP1 and Pak1 in innervated TA muscle (In) and 16h or 7 days after denervation (De). Protein levels are normalized to actinin and to innervated muscle. (**D**) Western Blot analysis of CtBP1 and Pak1 in innervated *soleus* muscle (In) and 2 days or 3 weeks after denervation (De). Protein levels are normalized to actinin and to innervated muscle. All values are mean ± s.d.; n=3 biological replicates per group (B), 3 and 4 muscles per group (C for 16h and 7 days), 4 and 3 muscles per group (D for 2 days and 3 weeks); **$p<0.01$ ***$p<0.001$; one-way ANOVA with Tukey's post-hoc (B) and Student's t-test (C, D).

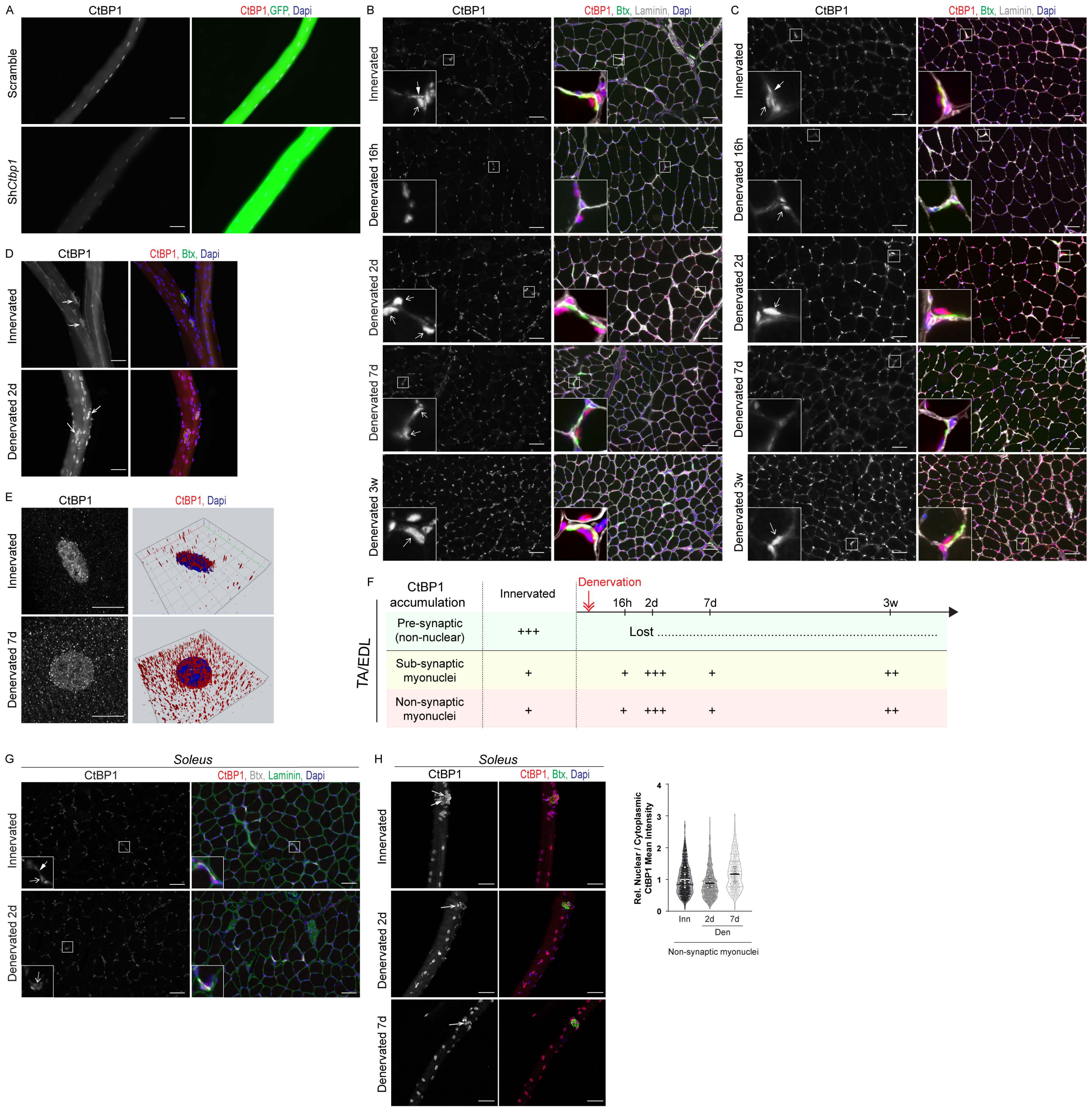

Fig. S2. CtBP1 accumulates in myonuclei of TA/EDL muscles 2 days after denervation. (**A**) Staining of single EDL fibers with CtBP1 antibody after AAV-sh*Ctbp1* (AAV9-GFP-U6-*mCtbp1*-shRNA) infection confirms the specificity of CtBP1 antibody. Scale bar, 50µm. (**B** and **C**) Immunostaining of innervated and denervated (16h to 3 weeks) TA sections with rabbit (B) and mouse (C) CtBP1 antibodies shows transient accumulation of CtBP1 in non- and sub- (open arrows) synaptic myonuclei 2 days and 3 weeks after denervation. Non-nuclear CtBP1 staining is visible at the NMJ region of innervated muscles (arrows). Scale bar, 50µm. (**D**) Immunostaining of innervated and denervated (2 days) single EDL fibers with mouse CtBP1 antibody shows transient accumulation of

CtBP1 in non- and sub- (open arrows) synaptic myonuclei 2 days after denervation. Scale bar, 50µm. (**E**) Enlarged view and 3D reconstruction of nuclear CtBP1 staining in innervated and denervated (7 days) TA muscles. Scale bar, 10µm. (**F**) Dynamic changes in CtBP1 accumulation after denervation in TA/EDL muscles. (**G-I**) Immunostaining of *soleus* muscle sections (G) and fibers (H) shows similar CtBP1 accumulation in non- and sub- (open arrows) synaptic myonuclei in innervated and denervated muscles. Quantification of the ratio of mean CtBP1 fluorescence intensity in non-synaptic myonuclei relative to cytoplasmic intensity, normalized to innervated muscle, is shown in I for isolated fibers. Small dots represent individual nuclei. Large dots indicate the mean ratio per independent muscle (biological replicate), which was used for statistical analysis. Values are mean ± s.d.; n=7/4/3 independent muscles. Scale bar, 50µm.

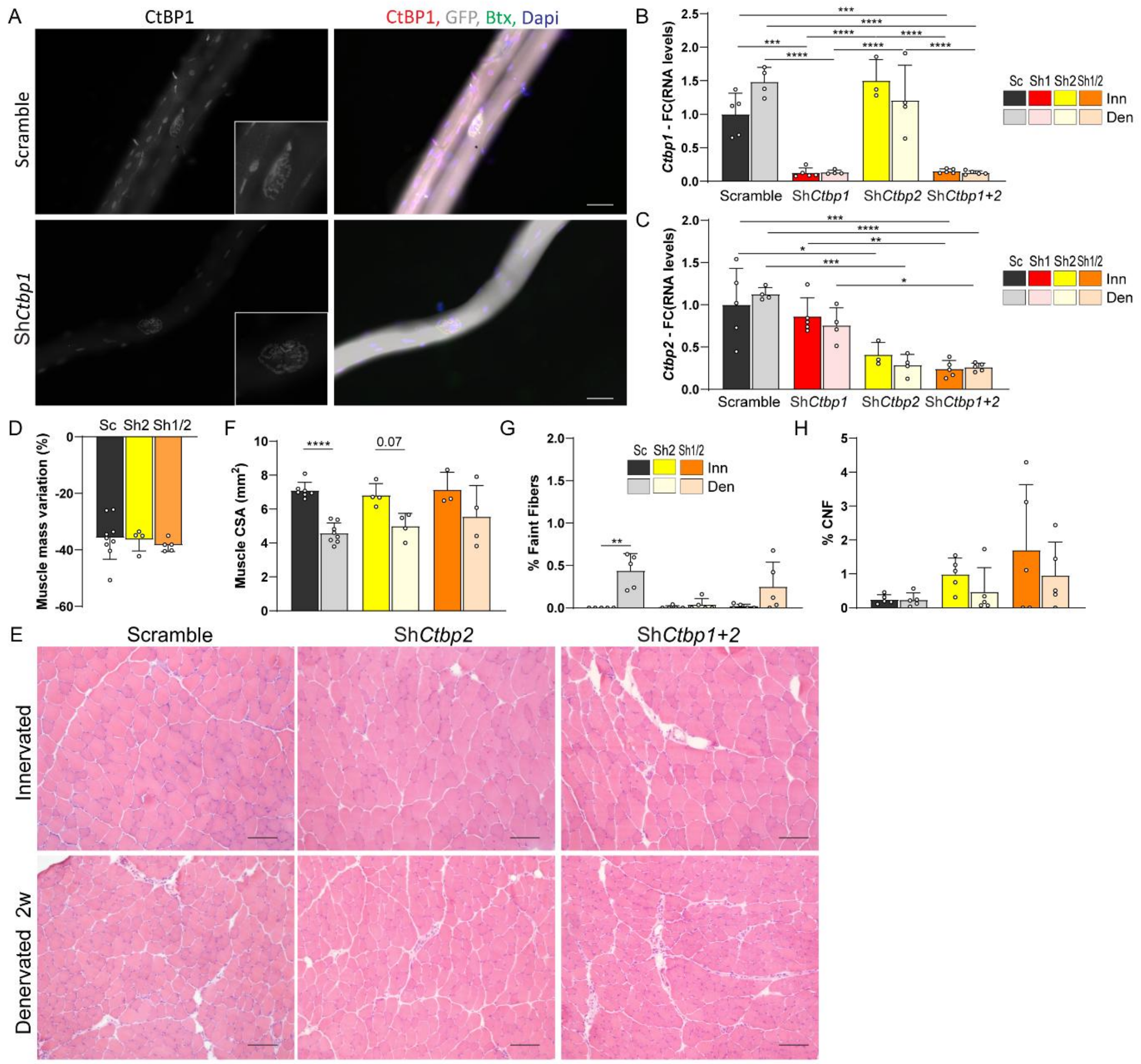

Fig. S3. *Ctbp2* knockdown does not affect *Ctbp1* expression or muscle histology. (**A**) Immunostaining with CtBP1 antibody of innervated single EDL fibers shows loss of CtBP1 staining in the muscle fiber, but not in the pre-synaptic compartment, after infection with AAV-sh*Ctbp1*. Scale bar, 50µm. (**B** and **C**) mRNA levels of *Ctbp1* (B) and *Ctbp*2 (C) in innervated (Inn) and denervated (Den, 2 weeks) TA muscles infected with AAV-*shCtbp1* (Sh1), -sh*Ctbp2* (Sh2), -sh*Ctbp1* and -sh*Ctbp2* (Sh1/2), or -shScramble (Sc). Levels are relative to *Tbp* mRNA and to Scramble innervated. (**D**) Mass variation after 2 weeks of denervation for TA muscles injected with AAV-*shCtbp2* (Sh2), AAV-*shCtbp1* and -*shCtbp2* (Sh1/2) or -shScramble (Sc). (**E**) H&E staining of innervated and 2-week-denervated TA muscles injected with AAV-*shCtbp2*, or AAV-*shCtbp1* and -*shCtbp2,* or -shScramble. Scale bar, 100µm. (**F**) Cross sectional area (CSA) of innervated and denervated (2 weeks) TA muscles injected with AAV-*shCtbp2* (Sh2), AAV-*shCtbp1* and -*shCtbp2* (Sh1/2) or -shScramble (Sc). (**G** and **H**) The proportion of abnormally faint fibers (G) and of centronucleated fibers (CNF, H) is unchanged in innervated and denervated TA muscles injected with AAV-*shCtbp2* (Sh2), AAV-*shCtbp1* and -

shCtbp2 (Sh1/2), compared to Scramble (Scr). All values are mean ± s.d.; n=5Sc/Sh1/Sh1+2 (Inn); 4Sc/Sh1/Sh2 (Den); 3InSh2; 5Sh1+2Den (B, C), 4Sh2/5Sh1+2 (D), 4 (F, except Sh1+2Inn, n=3), 5 (G, H) muscles per group; *p<0.05, **p<0.01, ***p<0.001, ****p<0.0001; two-way ANOVA with Tukey's post-hoc.

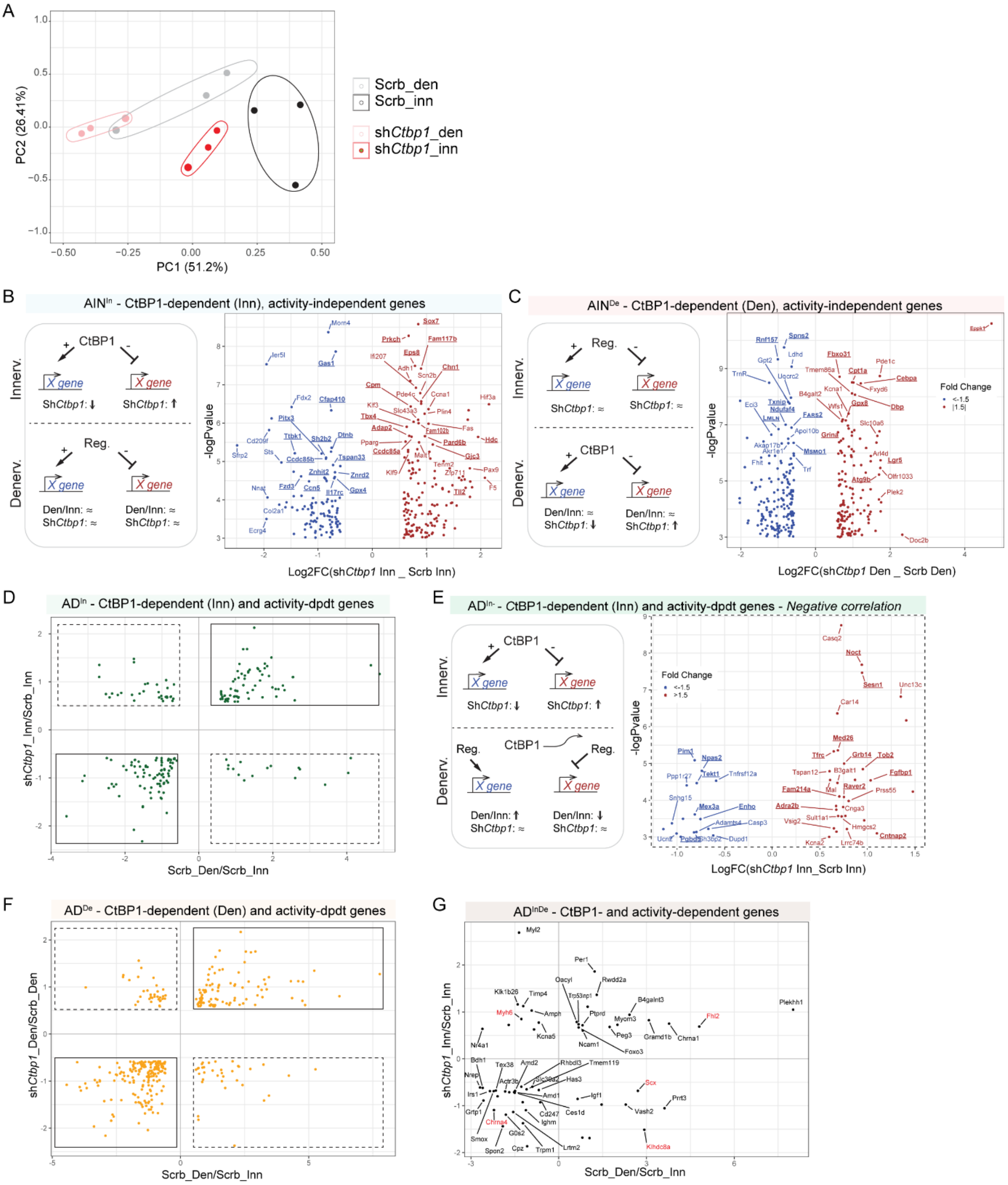

Fig. S4. Transcriptomic analyses of *Ctbp1*-silenced TA muscle 2 days after denervation. (**A**) Principal component analysis (PCA) showing separation of innervated (Inn) vs. denervated (Den), and AAV-sh*Ctbp1* vs. –shScramble groups. (**B** and **C**) *Ctbp1* knockdown alters the expression of activity-independent (AIN) genes in innervated (B) or denervated (C) muscles. Volcano plots show genes DE in AAV-sh*Ctbp1* innervated (B) or denervated (C) muscles (*vs*. AAV-Scrb). (**D** and **E**) *Ctbp1* knockdown affects the expression of activity-dependent genes only in innervated muscle (AD^{In}), with positive (full line) or negative (dotted line) correlation with the denervation effect. The volcano plot in E shows genes DE in AAV-sh*Ctbp1* innervated muscles (*vs*. AAV-Scrb innervated), with negative

correlation with denervation-induced changes. (**F**) *Ctbp1* knockdown affects the expression of activity-dependent genes only in denervated muscle (AD^{In}), with positive (full line) or negative (dotted line) correlation with the denervation effect. (**G**) Scatter plot showing activity-dependent genes DE in both AAV-sh*Ctbp1* innervated muscles (*vs*. AAV-Scrb innervated) and AAV-sh*Ctbp1* denervated muscles (*vs*. AAV-Scrb denervated). The plot shows the correlation between the effect of denervation and of AAV-sh*Ctbp1* in innervated muscle. Only some genes (red) show an inverse correlation in denervated muscle (see Fig. 5G). n=3 mice per group.

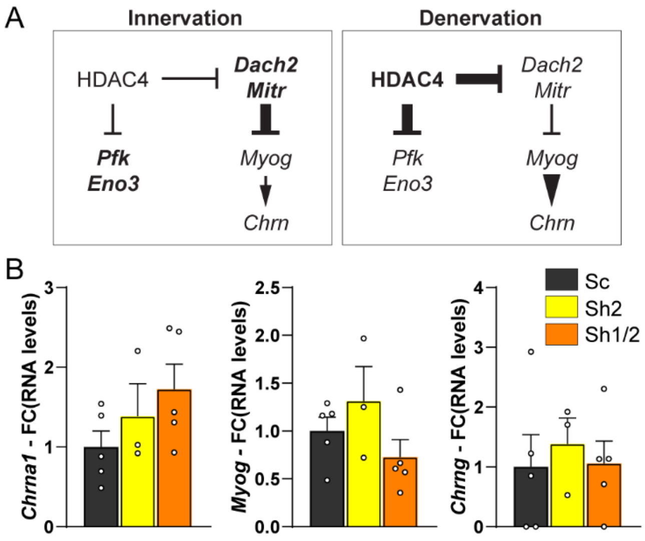

Fig. S5. ***Ctbp2* knockdown does not alter synaptic gene expression.** (**A**) HDAC4-dependent regulation of metabolic and synaptic genes in innervated and denervated conditions. (**B**) mRNA levels of *Chrna1*, *Myog* and *Chrng* after AAV-shScramble (Sc), -sh*Ctbp2* (Sh2), or -sh*Ctbp1* and -sh*Ctbp2* (Sh1/2) injection in innervated or denervated TA muscles. Levels are relative to *Tbp* mRNA and normalized to AAV-shScramble innervated muscle. All values are mean ± s.d.; n=5Sc/ Sh1/2, 3Sh2 (B) muscles per group.

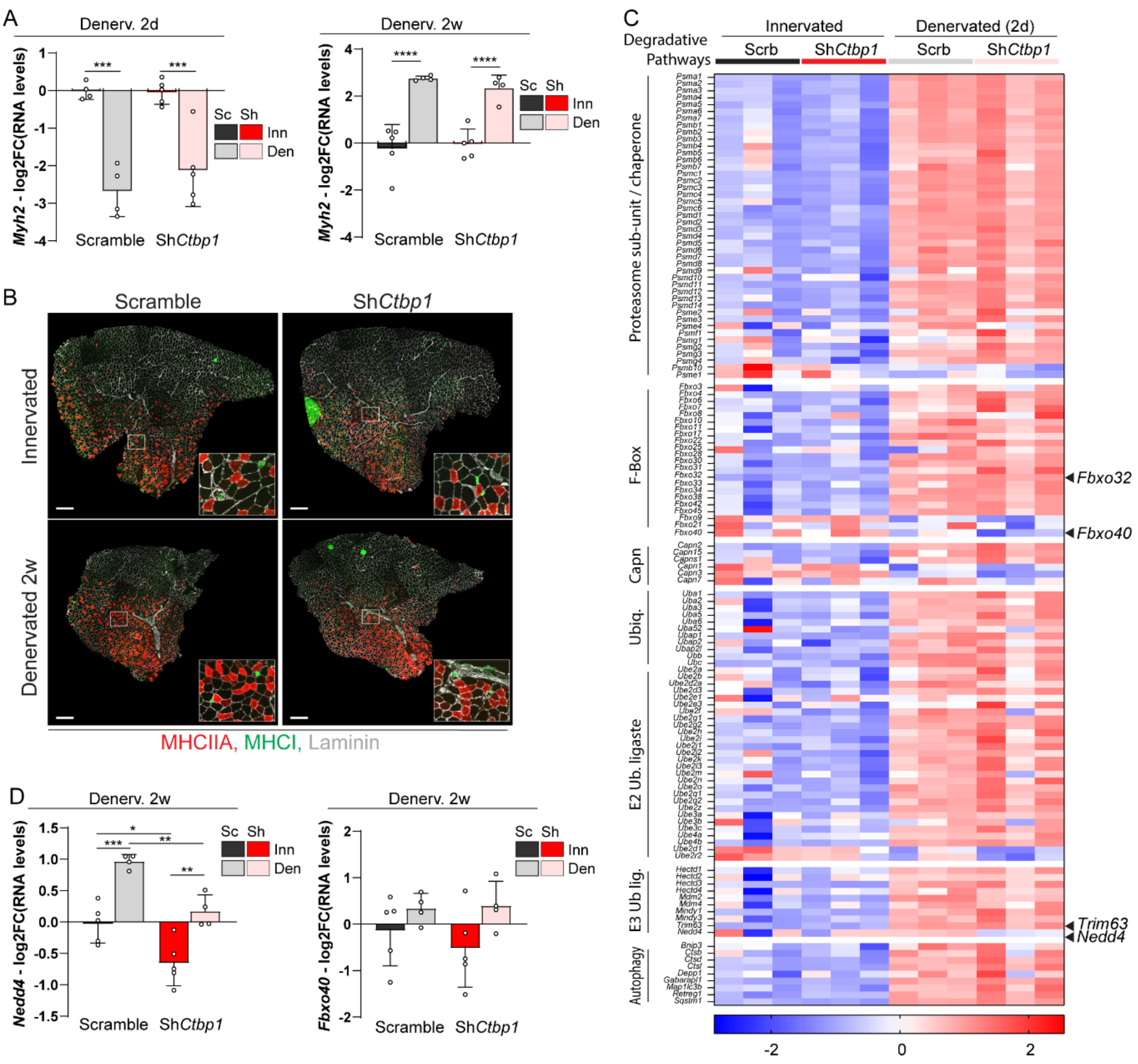

Fig. S6. *Ctbp1* knockdown does not affect genes linked to muscle atrophy. (**A**) mRNA levels of *Myh2,* encoding myosin heavy chains IIA after AAV-sh*Ctbp1* (Sh) or -shScramble (Sc) injection in innervated (Inn) and 2-day- and 2-week-denervated (Den) TA muscles. Levels are relative to *Tbp* mRNA and normalized to AAV-shScramble innervated muscle. (**B**) Immunostaining for type IIA and type I myosin heavy chains (MHC) and laminin, of innervated and 2-week-denervated TA muscles infected with AAV-sh*Ctbp1* or -shScramble. Scale bar, 500µm. (**C**) Heatmap of z-scores computed based on log2FC of RNAseq counts for genes encoding proteins involved in proteolysis, in innervated and denervated, AAV-sh*Ctbp1* and -shScramble muscles. (**D**) mRNA levels of *Nedd4* and *Fbxo40* after AAV-sh*Ctbp1* (Sh) or -shScramble (Sc) injection in innervated (Inn) and 2-week-denervated (Den) TA muscles. Levels are relative to *Tbp* mRNA and normalized to AAV-shScramble innervated muscle. All values are mean ± s.d.; n=4Sc/5Sh (A, 2d); 5Inn/4Den (A, 15d and D) muscles per group; *$p<0.05$, **$p<0.01$, ***$p<0.001$, ****$p<0.0001$; two- way ANOVA with Tukey's post-hoc.

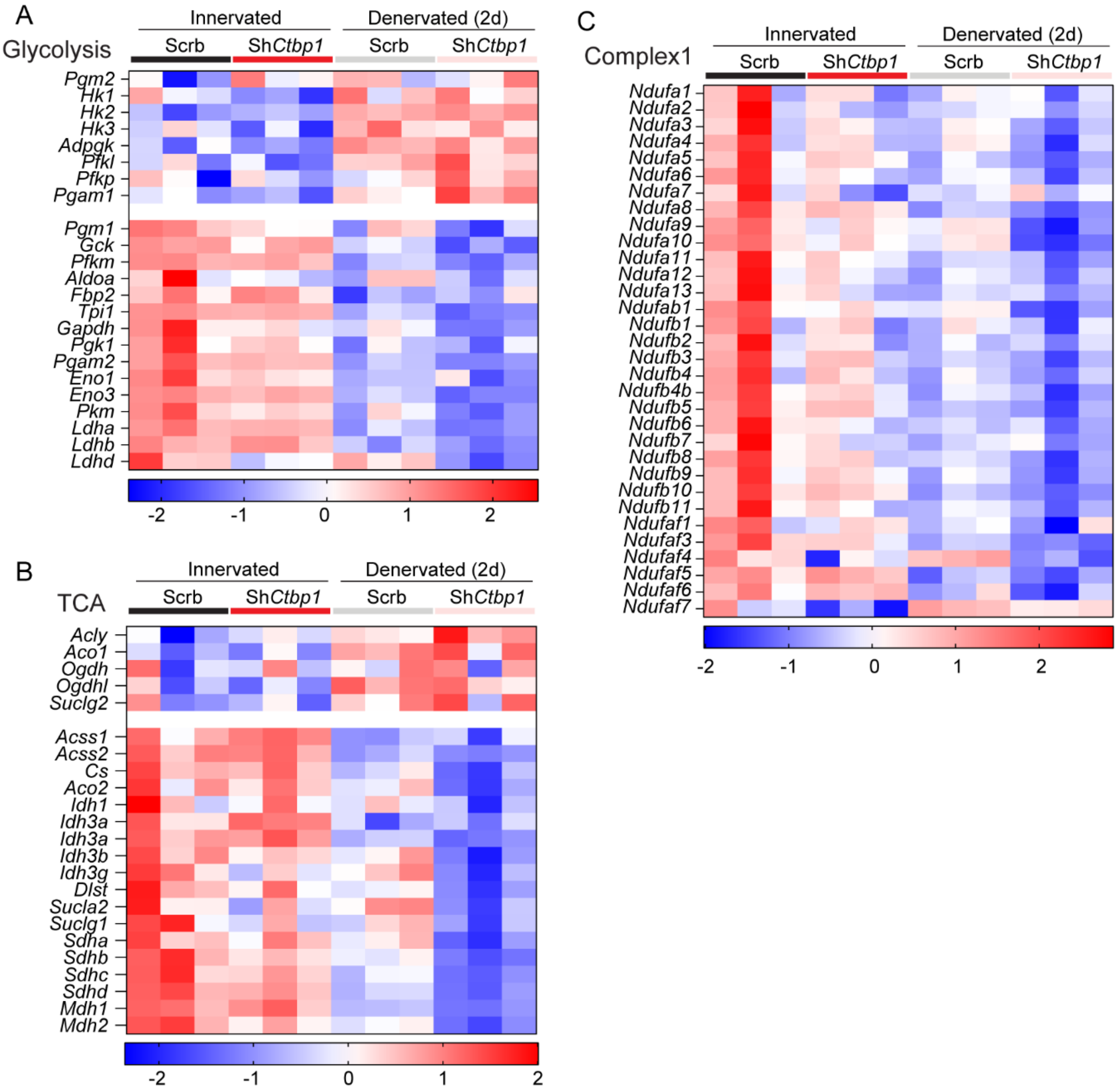

Fig. S7. *Ctbp1* knockdown exacerbates the effect of denervation on metabolic genes. (A-C) Heatmap of z-scores computed based on log2FC of RNAseq counts for nuclear genes encoding proteins involved in glycolysis (A), in tricarboxylic acid (TCA) cycle (B), and in respiratory chain complexes I (C), in innervated and denervated, AAV-sh*Ctbp1* and -shScramble muscles.

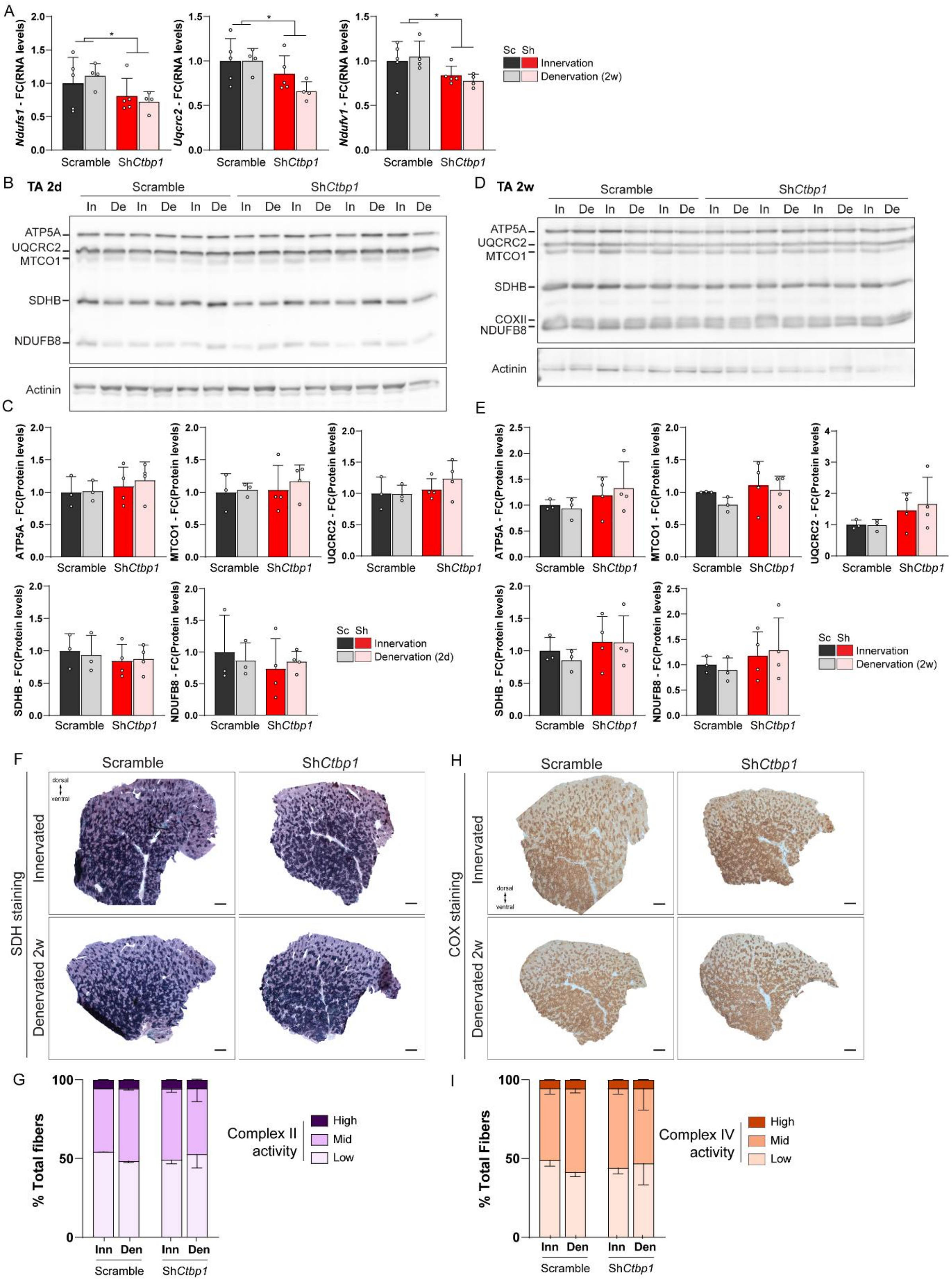

Fig. S8. *Ctbp1* knockdown does not change protein levels of respiratory chain complexes. (A) mRNA levels of genes encoding components of the respiratory complex I (*Ndufs1, Ndufv1*) and III (*Uqcrc2*), after AAV-sh*Ctbp1* (Sh) or -shScramble (Sc) injection in innervated (Inn) and 2-week-denervated (Den) TA muscles. Levels are relative to *Tbp* mRNA and normalized to AAV-shScramble

innervated muscle. (**B-E**) Protein levels of OXPHOS proteins from innervated (In) and 2-day- (B, C) or 2-week- (D, E) denervated (De) TA muscles infected with AAV-sh*Ctbp1* (Sh) or -shScramble (Sc). Protein levels are normalized to actinin and relative to Scramble innervated. (**F-I**) SDH (F) and COX (H) staining of innervated and 2-week-denervated TA muscles injected with AAV-sh*Ctbp1* or -shScramble. The proportion of fibers with high-, mid- and low-intensity SDH and COX staining is given in G and I, respectively. Scale bar, 500µm. All values are mean ± s.d.; n=5Inn/4Den (A), 3Sc/4Sh (B-E), 3 (F-I) muscles per group; *p<0.05; two-way ANOVA with Tukey's post-hoc.

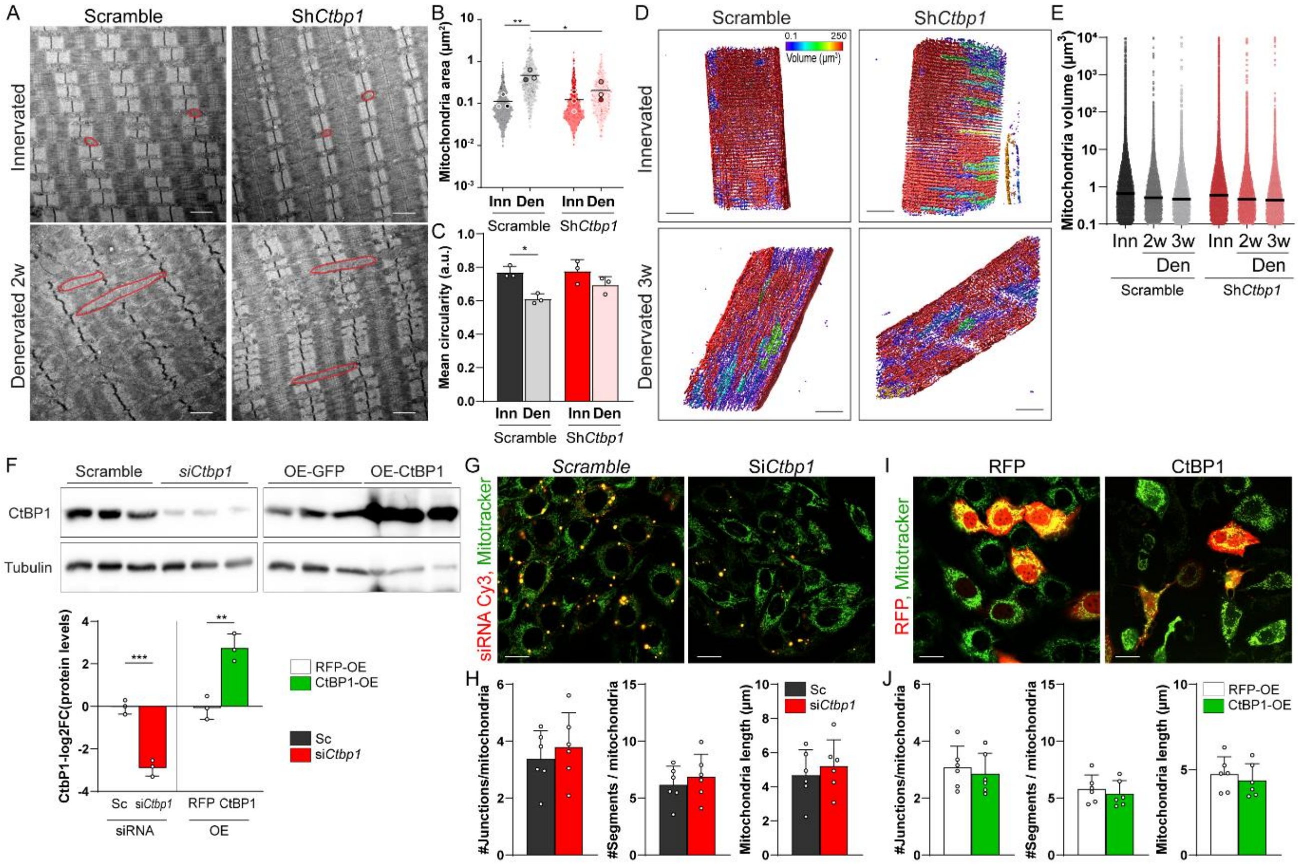

Fig. S9. ***Ctbp1* knockdown affects mitochondria network in mature muscle fibers.** (**A-C**) Electron microcopy of innervated and 2-week-denervated TA muscles injected with AAV-sh*Ctbp1* or -shScramble shows elongated mitochondria after denervation. Mitochondria area is given in B; mean circularity of mitochondria is given in C. Scale bar, 1µm. (**D** and **E**) Imaris 3D reconstitution of mitochondria network in innervated and 3-week-denervated TA muscles injected with AAV-sh*Ctbp1* or -shScramble, with color code corresponding to mitochondria volume. Original pictures are given in Fig. 8E. The distribution of mitochondria volume is given in E. (**F**) Protein levels of CtBP1 in C2C12 myoblasts transfected with siRNA against *Ctbp1* or CtBP1-overexpressing plasmids. (**G-J**) Morphology analysis of mitochondria labelled with Mitotracker Green FM in C2C12 myoblasts knocked-down for *Ctbp1* (cotransfected with siRNA-Cy3; G, H) or overexpressing CtBP1 (cotransfected with RFP plasmid; I, J). The length of mitochondria segments, their number per mitochondria and the number of segment junctions per mitochondria are given in H and J. Control cells are transfected with scramble siRNA-Cy3 (Sc) or RFP-overexpressing plasmids (RFP-OE). All values are mean ± s.d.; n=3 muscles per group (B, C); 3 biological replicates per group (F); 6 independent cell culture fields (H, J), *p<0.05; **p<0.01; ***p<0.001; two- way ANOVA with Tukey's post-hoc (B, C), Student's t-test (F).

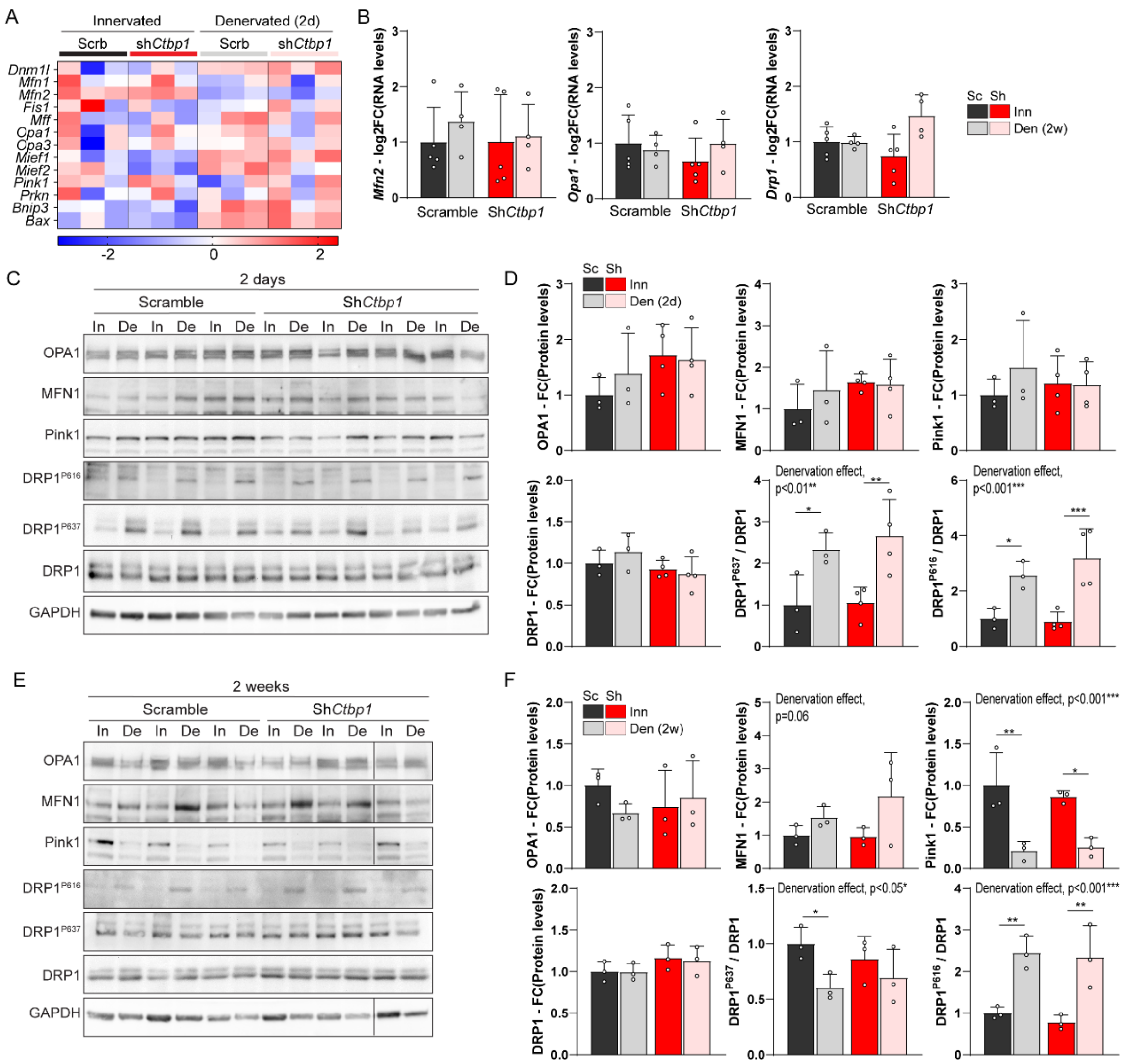

Fig. S10. *Ctbp1* knockdown does not alter levels of key mitochondria regulators. (**A**) Heatmap of z-scores computed based on log2 fold change of RNAseq counts for genes encoding proteins involved in mitochondria dynamics, in innervated and 2-day-denervated, AAV-sh*Ctbp1* and -shScramble muscles. (**B**) mRNA levels of genes encoding *Mfn2, Opa1* and *Drp1* after AAV-sh*Ctbp1* (Sh) or -shScramble (Sc) injection in innervated (Inn) and 2-week-denervated (Den) TA muscles. Levels are relative to *Tbp* mRNA and normalized to AAV-shScramble innervated muscle. (**C-F**) Levels of proteins involved in mitochondria fusion/fission (OPA1, MFN1 and DRP1) and mitophagy (PINK1) in innervated (Inn) and 2-day- (C, D) or 2-week- (E, F) denervated (Den) TA muscles, after AAV-sh*Ctbp1* (Sh) or -shScramble (Sc) injection. The vertical line indicates that samples were run on the same gel but were not contiguous. Quantification of protein levels is given in D and E. All values are mean ± s.d.; n=3 muscles per group (A), 5Inn/4Den muscles per group (B), 3Scr/4Sh muscles per group (D), 3Scr/3Sh muscles per group (F); *p<0.05; **p<0.01; ***p<0.001; two- way ANOVA with Tukey's post-hoc (B, D, F).

Supplementary Table1

Gene name	Forward primer	Reverse primer
Ctbp1-L	CTGGGCGTCCGACCTCCCATC	TCAGGATAGGCATCTCCACTG
Ctbp1-S	AATTCATGGTCGTGGAAACC	TCAGGATAGGCATCTCCACTG
Ctbp1-Pan	AGAGACCTTGGGCATCATTG	CCGCTCGATTCCATCAGATA
Pak1	ACACGGTTCGAGAAGATTGG	TCCCTCATGACCAGGATCTC
Myog	CACTCCCTTACGTCCATCGT	CAGGGCTGTTTCTGGACAT
Chrna1	TCCCTTCGATGAGCAGAACT	GGGCAGCAGGAGTAGAACAC
Chrng	GTGTCTTCGAGGTGGCTCTC	ACAGAGATGGAGCAGGAGGA
Chrne	TTCCCCTTTGACTGGCAGAA	AAAAGCTGCCGTGTCAATGT
Hdac4	CAGACAGCAAGCCCTCCTAC	AGACCTGTGGTGAACCTTGG
Dach2	CCAGCTCAAATCCCAGTCAT	CGCAGTTCCTTCTTTTCCTG
Mitr	CCACCTTGAAGAAGCAGAGG	TGGTGTCCTTAGAGGCTGCT
Pfk	GATGCAAGGACTTCCGAGAG	GCTCCACTCTGAACGGAAAG
Mse	GGGAGATGACCTCACGGTAA	TTACAGGCCTGGATGGACTC
Myh2	ACAAATCTATCCAAGTTCCG	TTCGGTCATTCCACAGCATC
Myl2	CGTGTTCCTCACGATGTTTG	CCTCTCTGCTTGTGTGGTCA
Myh4	CAGATGAAAAGGTGGCCATT	CTTCCCTTTGCTTTTGCTTG
Mfn2	GCACTTTGTCACTGCCAAGA	TGTGTTCCTGTGGGTGTCTT
Opa1	GAAGGACGACAAAGGCATCC	CCCGTGGTAGGTGATCTTGT
Drp1	TGCCTCAGATCGTCGTAGTG	GAAACGTGGACTAGCTGCAG

Supplementary references

1. Cocchiararo I, Cornut M, Soldati H, Bonavoglia A, Castets P. Back to basics: Optimization of DNA and RNA transfer in muscle cells using recent transfection reagents. Exp Cell Res. 2022;421(2):113392.

2. Li D, Hsu S, Purushotham D, Sears RL, Wang T. WashU Epigenome Browser update 2019. Nucleic Acids Res. 2019;47(W1):W158–W65.

3. Li D, Purushotham D, Harrison JK, Hsu S, Zhuo X, Fan C, et al. WashU Epigenome Browser update 2022. Nucleic Acids Res. 2022;50(W1):W774–81.
